# Structural Elucidation of a Polypeptoid Chain in a Crystalline Lattice Reveals Key Morphology-Directing Role of the N-Terminus

**DOI:** 10.1021/acsnano.2c12503

**Published:** 2023-02-23

**Authors:** Tianyi Yu, Xubo Luo, David Prendergast, Glenn L. Butterfoss, Behzad Rad, Nitash P. Balsara, Ronald N. Zuckermann, Xi Jiang

**Affiliations:** † Materials Sciences Division, Lawrence Berkeley National Laboratory, Berkeley, California 94720, United States; ‡ Department of Chemical and Biomolecular Engineering, University of California, Berkeley, California 94720, United States; § Molecular Foundry, Lawrence Berkeley National Laboratory, Berkeley, California 94720, United States; ⊥ Center for Genomics and Systems Biology, New York University, Abu Dhabi, United Arab Emirates

**Keywords:** self-assembly, peptoid crystallization, protein-mimetic materials, molecular packing, cryo-TEM, MD simulation

## Abstract

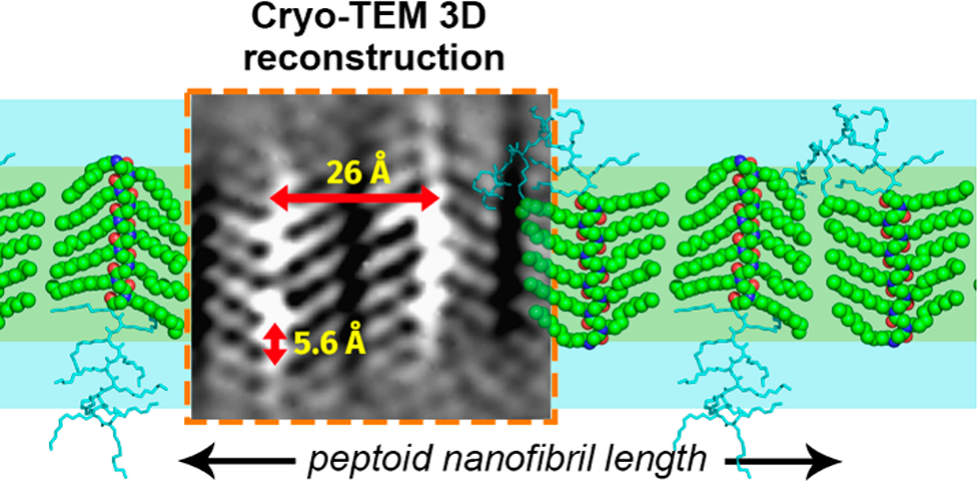

The ability to engineer synthetic polymers with the same structural precision as biomacromolecules is crucial to enable the *de novo* design of robust nanomaterials with biomimetic function. Peptoids, poly(*N*-substituted) glycines, are a highly controllable bio-inspired polymer family that can assemble into a variety of functional, crystalline nanostructures over a wide range of sequences. Extensive investigation on the molecular packing in these lattices has been reported; however, many key atomic-level details of the molecular structure remain underexplored. Here, we use cryo-TEM 3D reconstruction to directly visualize the conformation of an individual polymer chain within a peptoid nanofiber lattice in real space at 3.6 Å resolution. The backbone in the *N*-decylglycine hydrophobic core is shown to clearly adopt an extended, all-*cis*-sigma strand conformation, as previously suggested in many peptoid lattice models. We also show that packing interactions (covalent and noncovalent) at the solvent-exposed N-termini have a dominant impact on the local chain ordering and hence the ability of the chains to pack into well-ordered lattices. Peptoids in pure water form fibers with limited growth in the *a* direction (<14 molecules in width), whereas in the presence of formamide, they grow to over microns in length in the *a* direction. This dependence points to the significant role of the chain terminus in determining the long-range order in the packing of peptoid lattices and provides an opportunity to modulate lattice stability and nanoscale morphology by the addition of exogenous small molecules. These findings help resolve a major challenge in the *de novo* structure-based design of sequence-defined biomimetic nanostructures based on crystalline domains and should accelerate the design of functional nanostructures.

## Introduction

The biological functionalities of biomacromolecules (e.g., proteins, DNA, etc.) are strongly coupled with their self-assembled hierarchical structures, and their assembly information is encoded within the monomer sequence.^1,2^ Numerous efforts have been devoted to develop synthetic materials with similar hierarchical complexity and improved stability and performance using defined sequences of biomimetic building blocks.^3,4^ Peptoids are a promising class of peptide-mimetic polymer with side chains appended to the backbone amide nitrogen rather than the α-carbon.^5^ Devoid of extensive −NH hydrogen bond donors and chiral centers along the backbone, the structure and function of peptoids are governed by the identity of side chains and their sequence. Thus, peptoids are an ideal platform to explore the impact of monomer sequence on their ability to fold and assemble into defined nanostructures.^6^ The solid-phase submonomer synthesis (SPSS) method developed by Zuckermann *et al*. enables the precise control over the monomer sequence in peptoids, providing a powerful tool for systematic investigation of the impact of the molecular structure on their self-assembly behaviors.^7^ Sequence-defined peptoids with varying crystallizable side chains have been designed to form diverse supramolecular nanostructures in solution such as crystalline nanotubes,^68^ nanoribbons,^8^ nanosheets,^8−9^ nanobrushes,^10^ and nanostars.^12^

Despite the diversity of these nanoscale morphologies, the molecular conformation and packing geometry of crystal lattices of many peptoid nanostructures share an extended, all-*cis*-backbone conformation packed into roughly rectangular cores.^8,10,11−15^ The distinctive planar conformation of the extended *cis*-backbone results in a rectangular molecular shape, which prefers to adopt lamellar morphologies in crystalline diblock co-polypeptoid lattices over an unusually broad compositional range (ϕ = 0.11–0.65).^16^ However, previous studies on peptoid lattice structure which were based on X-ray scattering and electron microscopy are of limited resolution, revealing only the molecular packing geometry,^9,13−15,17^ but not the atomic details of the polymer conformation. Although many of the atomic details can be inferred from MD simulation, there are a number of unanswered questions in peptoid nanostructures which require higher-resolution experimental methods to provide further atomic detail. For example, small changes in the N-terminal chemistry have been shown to dictate liquid crystallinity versus crystallinity.^19^ Determining the atomic details in peptoid nanostructures can also help identify the molecular bases of unusual assembly morphologies. For example, distinct from other reported nanostructures with crystalline cores,^8,10,13−15^ an amphiphilic diblock co-polypeptoid, poly(*N*-decylglycine)-*block*-poly(*N*-2-(2-(2-methoxyethoxy)ethoxy)ethylglycine) (Ndc_9_-Nte_9_)^18^ was reported to form nanotubes with a high degree of chain curvature and no central hydrophobic core.

High-resolution cryogenic transmission electron microscopy (cryo-TEM) has been a powerful technique for elucidating well-ordered biomolecular structures, such as helical fibrils, nanotubes and nanosheets, formed by peptides^20−25^ and proteins.^26−30^ Three-dimensional (3D) structures of peptide assemblies have been resolved to near-atomic precision by averaging the global structural information using helical reconstruction^31−33^ and electron crystallography.^34−36^ In contrast, the structure of crystalline peptoid nanoassemblies, have only been partially resolved experimentally. Peptoid nanosheets have been imaged using cryo-TEM,^9,17,37,38^ however, the 3D molecular conformation of an individual synthetic polymer chain in a crystalline polymer lattice has not yet been directly observed due to increased heterogeneity of their assemblies. Single-particle analysis (SPA) is an emerging powerful imaging technique to analyze the molecular details at atomic and near-atomic resolution of assembled nanostructures from natural biopolymer complexes (e.g., peptide assemblies,^39^ ribosomes,^40^ membrane proteins,^41,42^ viruses^43^) or synthetic organic^44^ and inorganic materials.^45,46^ It not only enables the 2D and 3D classification of inhomogeneity in the nanostructures^47,48^ but also allows for reference-free *ab initio* 3D reconstructions using sophisticated algorithms.^49,50^ Here, we apply cryo-TEM 3D reconstruction to directly visualize the spatial conformation of the peptoid chain in crystalline peptoid fibrils.

We chose to study our previously reported nanotubes (Ndc_9_-Nte_9_),^18^ which as mentioned above, have a proposed unusual packing morphology forming nanotubes with high chain curvature and the absence of a hydrophobic core. The 1D morphology of this material lends itself to analysis by cryo-TEM 3D reconstruction, as has been reported for peptide nanostructures.^1,51,52^ Herein, we revisit the assembly model of these nanotubes and explore their structure and packing in molecular detail. Interestingly, our higher-resolution study revealed that the previously reported nanotubes are in fact nonhollow nanofibers with similar molecular conformation and rectangular packing geometry as in several previously reported nanosheets.^8,9,13,17^ Furthermore, the 3D reconstructed electron density map provides the direct visual evidence of a peptoid in an all-*cis*-sigma strand conformation.

Since the molecular conformation in these nanofibers turns out to be highly homologous to nanosheets, we further sought to understand the molecular determinants of nanofiber versus nanosheet formation. We turned our attention to the N-terminus since it has been previously shown that small changes at the N-terminal amine can have major impact on peptoid structure and thermal properties.^19,51^ We modulated the chemical nature of the N-terminus region by varying end capping groups as well as by the addition of exogenous small molecules. Indeed, we found the N-terminus plays a structure-directing role in the formation of nanoscale sheets versus fibers.

In this contribution, using a combination of cryo-TEM 3D reconstructions, differential scanning calorimetry (DSC) analysis, molecular dynamic (MD) simulation, and quantum mechanics (QM) calculation, we determined the molecular conformation of a peptoid chain in a crystalline lattice at resolution of 3.6 Å. We further demonstrate that certain small molecules (e.g., urea or formamide) can stabilize the solvent-exposed N-terminal region of peptoid fibrils in water, and propagate an increase in the degree of order throughout the entire crystalline lattice. This finding elucidates how covalent and noncovalent interactions at the N-terminal nitrogen atom of a peptoid chain, directs the hierarchical assembly morphology into nanofibrils or nanosheets.

## Results and Discussion

### Peptoid Design

**Figure 1:**
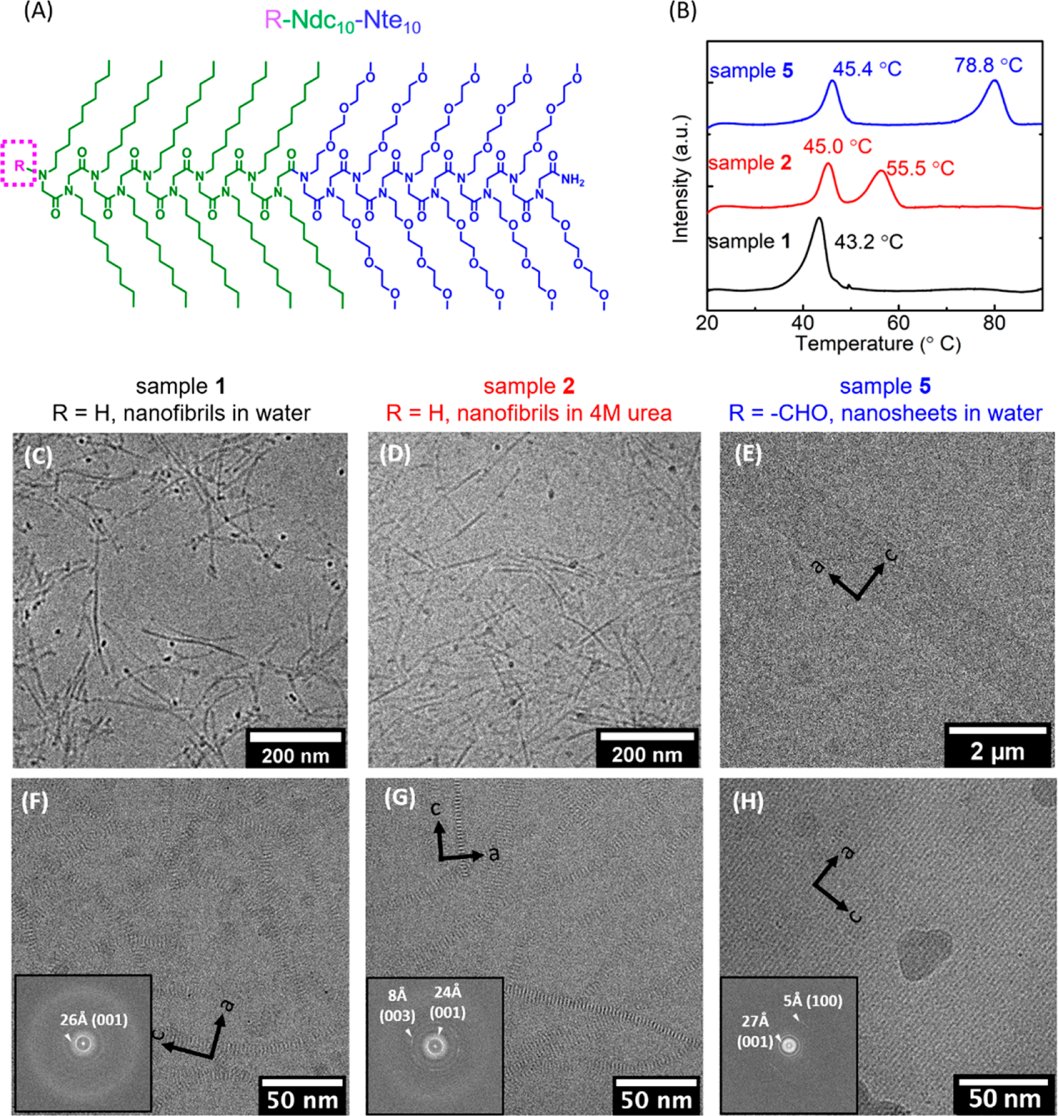
(A) Molecular structure of R-Ndc_10_-Nte_10_. (B) Solution DSC analysis of H-Ndc_10_-Nte_10_ in water (sample **1**), 4 M urea (sample **2**), and F-Ndc_10_-Nte_10_ nanosheets in water (where F = formyl) (sample **5**). Low-dose cryo-TEM micrographs of peptoid nanostructures embedded in ice at (C–E) low magnification and (F–H) high magnification of samples **1**, **2**, and **5**. In the high-magnification images (F–H), characteristic stripes are observed at *d* = 26 ± 1 Å. Crystallographic axes are indicated (black arrows). Dark areas in all images represent the electron dense regions. The fast Fourier transforms are shown in the insets in (F–H).

The co-polypeptoids used in this study are monodispersed diblock co-polypeptoids made from two monomer building blocks: a hydrophobic and crystallizable poly-*N*-decylglycine (Ndc) segment and a hydrophilic poly-*N*-2-(2-(2-methoxyethoxy)ethoxy) ethylglycine (Nte) segment. Previously we reported that, over three different chain lengths, this family of diblock peptoids formed crystalline nanotubes.^18^ Since that report, many additional studies have determined that poly(*N*-alkyl glycine) chains adopt a planar molecular conformation, and form rectangular lattices with the side chains displayed in an opposing manner.^9,37^ These studies prompted us to revisit the nanotube model and study these structures at much higher resolution. We focus here on two peptoids, Ndc_10_-Nte_10_, with and without an N-terminal capping group (Figure 1A). These molecules have an even number of hydrophobic side chains, and they form well-defined crystalline nanostructures which have increased crystallinity compared to previously reported Ndc_9_-Nte_9_ (which has an odd number of side chains) as determined by DSC (Figure S3 and Table S1). The shorter chain length of the 20mer was chosen to facilitate ease of synthesis, purification, structure determination, and computational modeling.

Surprisingly, the *N*-formyl-capped versus noncapped compounds form very different nanoscale morphologies upon evaporation of THF/water solutions (Table 1). H-Ndc_10_-Nte_10_ with a free N-terminus (which is protonated in the assembly conditions) forms similar 1D morphologies as previously reported for Ndc_9_-Nte_9_ nanotubes (Figure 1C),^18^ whereas F-Ndc_10_-Nte_10_ with a N-terminal formyl group forms large nanosheets (Figure 1E), consistent with the formation of nanosheets with other N-terminal acyl capping groups with sp^2^ hybridization (e.g., acetyl, chloroacetyl, and iodoacetyl) as previously reported.^17,19,37^ This points to the powerful structure-directing effect of the N-terminus. Prior studies have found that small changes at the peptoid N-terminus can result in significant changes to peptoid conformation^53^ or melting behavior.^19^ To better understand the structure-determining role of the N-terminus in these Ndc_10_-Nte_10_ nanostructures, we use single-particle cryo-TEM to probe the structural details at the near-atomic level.

**Table 1:** Solution Differential Scanning Calorimetry (Nano-DSC) and Cryo-TEM Characterization of the Nanofibrils and Nanosheets

						lattice spacing^c^	
entry	compound	solvent	morphology^a^	*T*_1_ (°C)	*T*_2_ (°C)	*c* spacing (Å)	*a* spacing (Å)
1	H-Ndc_10_-Nte_10_	water	fibrils	43.2	b	26	4.9
2		4 M urea in water	fibrils	45.0	55.5	26	4.9
3		4 M formamide in water	fibrils + small sheets	40.8	51.6	d	d
4		8 M formamide in water	sheets	46.9	72.6	26	4.9
5	F-Ndc_10_-Nte_10_	water	sheets	45.4	78.8	27	5.2
6	H-Nte_10_-Ndc_10_	water	sheets	40.6	76.7	d	d

a As determined by cryo-TEM reconstruction and AFM.

b No melting transition (*T*_2_) was observed.

c As determined by cryo-TEM reconstruction.

d Not determined.

### Nanofibril TEM 3D Reconstruction

**Figure 2:**
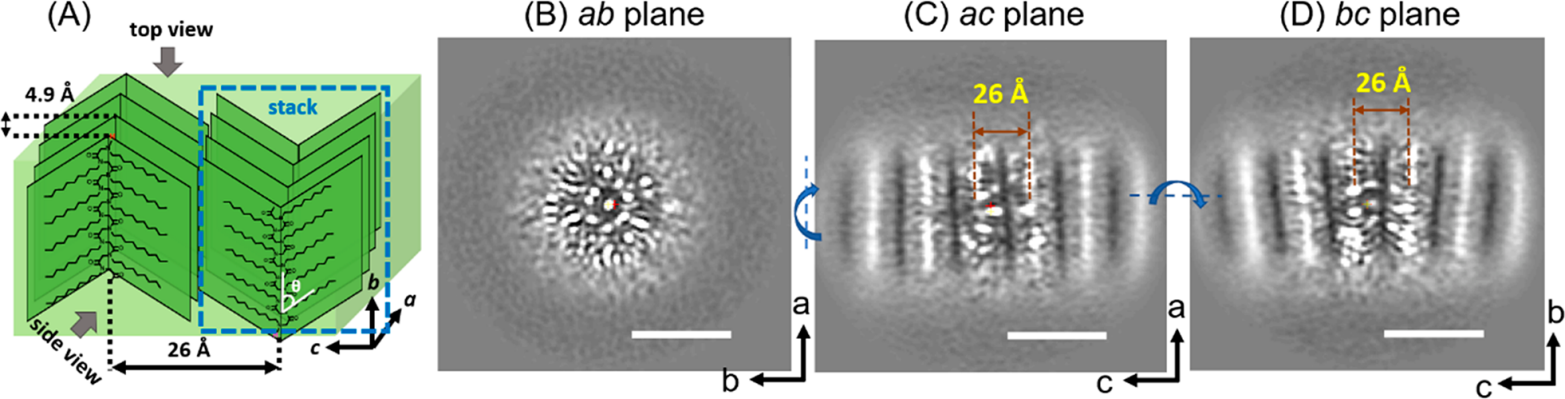
3D reconstruction of H-Ndc_10_-Nte_10_ nanofibrils in water (sample **1**) using cryo-TEM. (A) Schematic showing the molecular arrangement of the Ndc chains in the crystalline lattice. (B) *ab* cross section (end view), (C) *ac* cross section (top view), and (D) *bc* cross section (side view) of the nanofibril. Scale bar is 5 nm.

To elucidate the local impact of the N-terminus on chain-chain packing in the crystal lattice, a full understanding of the 3D chain packing of peptoids in the assembled H-Ndc_10_-Nte_10_ nanostructures (sample **1**) is necessary. The random orientations of the rod-like shapes in the assembly solution allow them to be imaged from varying angles under electron beam,^39^ which is difficult to accomplish with extended planar structures like nanosheets. A 3D reconstruction of the H-Ndc_10_-Nte_10_ crystalline nanostructures in water (sample **1**) was performed using low-dose cryo-TEM. In brief, a thin layer of the aqueous assembly solutions containing randomly oriented nanostructures, was frozen and 2D projections along the long axis of the rods were collected and processed using the SPA ab initio reconstruction method.^39−42^ The electron micrographs obtained were sorted and averaged to construct 3D electron density maps (see the Supporting Information for details). Three orthogonal cross section views of a H-Ndc_10_-Nte_10_ rods (sample **1**), which are obtained by stacking five slices of density map (0.7 Å each) along different directions in the 3D map, are displayed in Figure 2B–D.

The end view slice in Figure 2B reveals that the morphology is not a hollow tube as previously thought,^18^ but rather a fiber with a solid core, and a less bright outer layer. Based on analogy to the molecular packing of other *N*-alkyl peptoid nanosheets,^19,37^ we posit that the solid core is comprised of hydrophobic Ndc_10_ blocks, and the outer layer consists of hydrophilic Nte_10_ blocks exposed to water. We thus use the same lattice vector conventions in the following discussions. In an idealized lattice, each molecule is roughly planar, where the vector along the length of the peptoid backbone is defined as the *b* direction, and the vector emanating from the backbone to the tip of the side chain is defined as the *c* direction (Figure 2A). The two distinct cross sections through the length of the fiber both exhibit a predominant spacing of 26 ± 0.5 Å (Figure 2C,D). Fast Fourier transform analysis of the combined slices (Figure S8A) for one of the cross sections (Figure 2C) shows weak signals at 4.9 Å, which is close to the characteristic *a* chain spacing in the previously reported *cis*-sigma strand rectangular lattice in peptoid nanosheets.^9,17,19,37^ Even though the conformational and packing heterogeneity are greater than in the nanosheets,^19,37^ this feature is distinct as the top view (*ac* plane) of the lattice. Rotating 90° to the *bc* plane, which is the side view of the fiber not previously imaged in the nanosheet, in addition to the 26 ± 0.5 Å feature, we would expect to see a characteristic distance of 5.6 Å between every other decyl side chain. However, this spacing is not resolvable due to structural heterogeneity.

In both the top (Figure 2C) and side view slices (Figure 2D), the polymers are clearly packed into lamellar stacks, further indicating that the molecular packing consists of the chains in a rectangular lattice akin to a nanosheet as opposed to a tubular structure.^18,37^ These peptoid molecules pack face-to-face along the *a* direction (Figure 2A), which hereafter are referred to as “stacks” (Figure 1A). Each stack consists of only ∼ 12 molecules in the *a* direction, yet are able to grow to a length of hundreds of nanometers through side chain-to-side chain interactions along the *c* direction. Due to inherent heterogeneity of the sample (sample **1**), a detailed understanding of the molecular structure was not possible to resolve.

### Enhanced Chain Ordering with Addition of Exogenous Small Molecules

To explore the detailed chain packing of the nanofiber, a more ordered structure is desired for SPA 3D reconstruction. Hence, we sought an approach to enhance the chain ordering within the H-Ndc_10_-Nte_10_ nanofibers. Because N-terminal capping of the Ndc-Nte chains are enough to form extended nanosheets (Figure 1E) and not fibers,^37^ we aimed to make more subtle changes to the N-terminus. As an alternative to covalent modification, we explored if we could modulate lattice ordering by tuning the chemical environment of the free N-termini using exogenous small molecules. Urea and formamide, polar small molecules that contain both strong hydrogen bonding donors and acceptors, are commonly used to modulate the inter/intramolecular hydrogen bonding interactions in proteins/peptides.^54^ However, since peptoid lattices are not held together by networks of NH-O hydrogen bonds, we reasoned that these reagents may interact with the solvent-exposed free N-termini. Herein, we studied the ability of urea and formamide to modulate ordering of the fiber crystal lattice.

**Figure 3:**
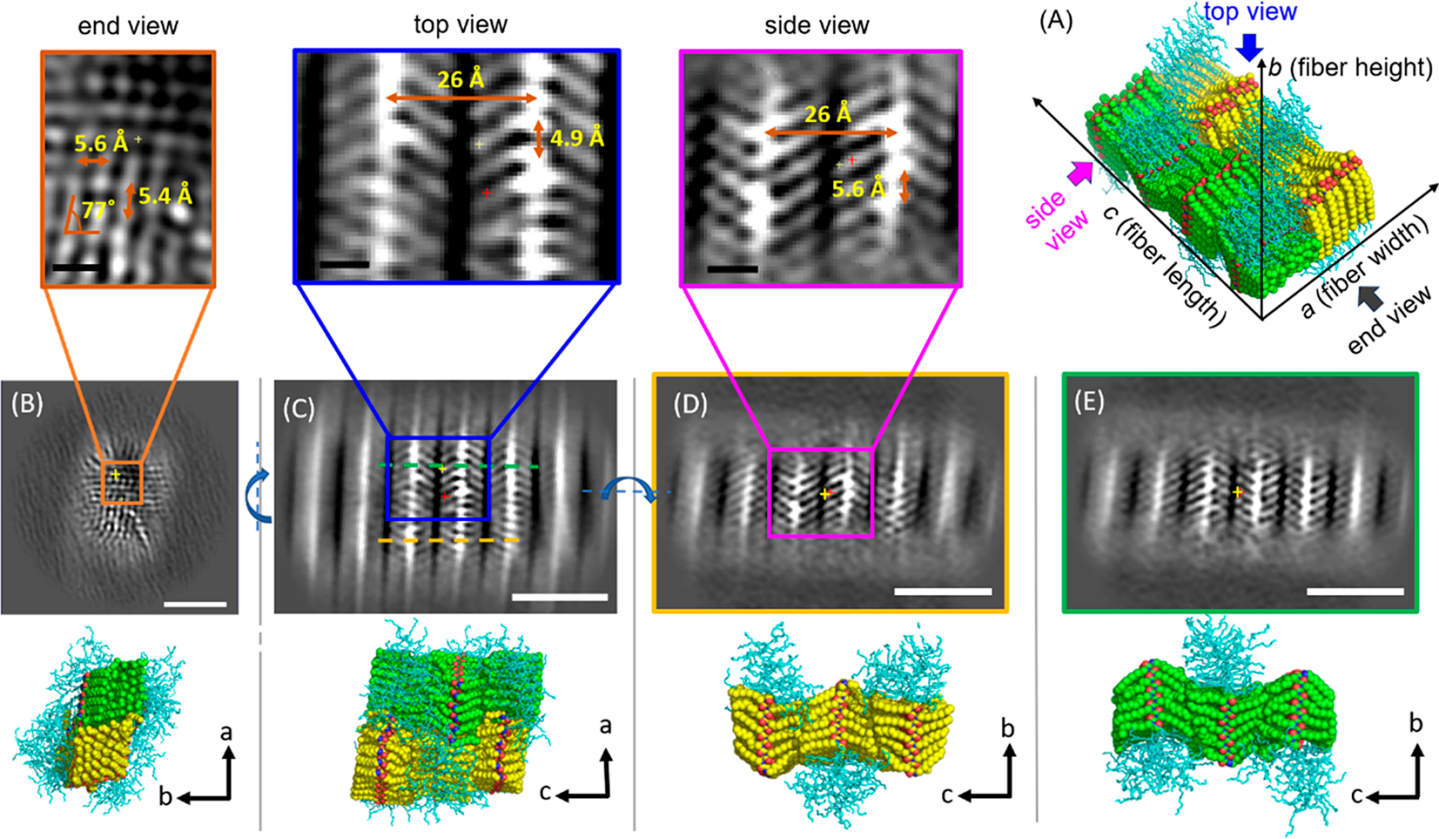
(A) Molecular representation showing the proposed peptoid packing geometry in the H-Ndc_10_-Nte_10_ nanofibrils in urea (sample **2**). (B–E) Slices from the TEM 3D reconstructions and MD simulation models in (B) end view (*ab* cross section), (C) top view (*ac* cross section), (D,E) side views (*bc* cross sections) of the nanofibrils. (C) Top view shows the fiber is composed of two identical segments with inverted symmetry marked in yellow and green, respectively. (D,E) Side view slices of these two segments projected from the positions marked with green and yellow dash lines in the top view slice in (C). Box regions show enlarged TEM slices with annotated lattice spacing information. The scale bar is 5 nm in (B–E) and 10 Å in the enlarged images. The thickness of each cross section slice is 3.5 Å in (B–E).

Cryo-TEM images of H-Ndc_10_-Nte_10_ assembled in the presence of 4 M urea also forms elongated nanofibers (sample **2**, Figure 1D,G). The crystallinity of the nanofibers were analyzed by DSC in both water (sample **1**) and urea solution (sample **2**, Figure 1B). Nanofibrils of H-Ndc_10_-Nte_10_ formed without urea (sample **1**) exhibited only one phase transition temperature (*T*_1_) at 43.2 °C (Figure 1B and Table 1), whereas two thermal phase transitions (*T*_1_ = 45.4 °C, *T*_2_ = 78.8 °C) were observed in F-Ndc_10_-Nte_10_ nanosheets (sample **5**, Figure 1B and Table 1). It has been previously shown that two thermal transitions in poly (*N*-alkyl glycines) corresponds to the presence of a mesophase between *T*_1_ and *T*_2_.^19^ Thus, we conclude that *T*_1_ is associated with losing the “tip-to-tip” interactions between the end −CH_3_ groups on the *n*-decyl side chains along the *c* direction, and *T*_2_ is from melting the face-to-face packing along the *a* direction upon heating and is also the order-to-disorder transition.^19^ Interestingly, H-Ndc_10_-Nte_10_ fibers grown from an aqueous urea solution (sample **2**) exhibit two thermal transitions like a nanosheet, with the second phase transition temperature (*T*_2_) at 55.5 °C, suggesting stronger face-to-face interchain packing along the *a* direction than without urea present. X-ray diffraction (XRD) analysis also suggests enhanced crystallinity with sharper diffraction peaks in the presence of urea. The full width at half-maximum (fwhm) of two peaks were analyzed: (001) represents the spacing along the fiber length (*c* dimension in Figure 3A), and (102) refers to the peak along the fiber width (*a* dimension in Figure 3A).^19^ The width of the (001) and (102) peaks of the H-Ndc_10_-Nte_10_ fibril (sample **1** in Figure S4 and Table S2) decreased 50.0 and 37.5%, respectively, with urea (sample **2** in Figure S4 and Table S2), consistent with the enhanced crystallinity. Since DSC and XRD analysis both revealed that H-Ndc_10_-Nte_10_ fibrils formed in 4 M urea (sample **2**) are more ordered than those formed in water (sample **1**), yet still form rod-like morphologies that lend themselves to cryo-TEM 3D reconstruction, we next focused on determining their high-resolution structures.

### Molecular Conformation and Packing in the Nanofiber Lattice

The molecular packing details of H-Ndc_10_-Nte_10_ nanostructure in 4 M urea aqueous solution (sample **2**) was resolved by the SPA 3D reconstruction. The molecular packing can be understood by viewing thin slices through the fiber along its three axes (Figure 3). Cross sections taken from the *ab* (end view in Figure 3B), *ac* (top view in Figure 3C), and *bc* (side view in Figure 3D,E) planes of the nanostructure are shown in Figure 3. The electron dense regions are brighter.

Distinct from the structure without urea (Figure 2C,D), the polymer side chain packing in both top (Figure 3C) and side (Figure 3D,E) views is significantly more ordered in the presence of urea. The spatial resolution (3.6 Å) is not high enough to elucidate individual atoms, but the spatial arrangement of individual chains in each crystalline stack and the heterogeneity of the structures in the fiber can be observed. The 3D reconstructed map also allows us to create and validate a 3D model, which is shown in Figure 3A, in order to explore the intermolecular interactions.

First, we consider the cross section in the *ab* plane (Figure 3B). It is noteworthy that this view of a peptoid lattice has never been directly visualized. This end view directly tells us the fibrils have a solid, crystalline hydrophobic core, and are clearly not hollow nanotubes as previously reported.^18^ The bright spots represent the tips of the ordered *n*-decyl side chains, the methyl groups, while the fuzzy outer layer that warps the crystalline core are the amorphous hydrophilic blocks exposed to water. The lattice packing of these terminal methyl groups in the enlarged image (shown in the orange box) exhibit a spacing between adjacent tips along the *a* direction of 5.4 ± 0.2 Å, and a spacing of 5.6 ± 0.1 Å in the *b* direction, with a tilt of 77° (Figure 3B). Although we would expect the backbones to stack directly on the top of each other, the side chains exhibit a slight offset in the *b* direction resulting in the observed tilt. This may be due to the inherent packing preference observed in alkyl chain lattices as exemplified by the decane lattice, where a tilt of 75° is observed.^55^

A top view (*ac* plane) of the fiber is shown in Figure 3C. The bright spots represent the glycine backbones and the bright arms emanating from the spots are *n*-decyl side chains. Three stacks of peptoid molecules can be clearly observed in the center region of top view. The outer stacks are blurry due to a round mask applied in SPA 3D reconstruction (see Experimental Section). It is observed that the width of the fibril in the *a* direction is limited to 12–14 chains. The stacks exhibit the same V-shaped arms with the side chains extending out on both sides of the backbones along the *c* direction as in previously reported nanosheets.^37,56^ We also observed the presence of two symmetrical crystalline domains with the arms of the V-shapes pointing toward to each other (i.e., < >) (Figure S19), as indicated in green and yellow, respectively, in the bottom molecular models (Figure 3C). The average spacing between the side chain methyl groups in the adjacent arms along the *a* direction was measured to be 4.9 ± 0.2 Å, and the spacing between two neighboring peptoid backbones along the *c* direction was 26 ± 0.5 Å (Figure 3C). Further analysis of the cross section in the *ac* plane (Figure 3C) reveals that the V-shaped arms in the two segments point toward the center of the fibril along the *a* axis. To understand the conformational difference of the two segments, one selected slice from each segment (marked in green/yellow dashed line) was rotated 90° along the *a* axis to obtain the side view cross sections (*bc* plane) in Figure 3D and 3E, respectively. Our 3D reconstruction provides a direct image of an individual peptoid backbone in position space. The distance between the adjacent repeating side chains along one side of the peptoid backbone (*b* direction) is 5.6 ± 0.1 Å in the first segment (yellow in Figure 3D), which is consistent with the measurement in *ab* plane (Figure 3B), indicating the peptoid molecules adopt a universal extended *cis*-configuration in the crystal lattice.^15^Figure 3E shows the molecular packings in the second segment (green) where the same characteristic distances are observed.

The combined information from top and side view images reveals the peptoid adopts an all-*cis* extended board-like chain packing geometry, nearly identical to the 3D chain packing in the F-Ndc_10_-Nte_10_ extended nanosheets (sample **5**, Figure 1H), indicating each fibril layer in the nanofibril is essentially a narrow strip of a nanosheet, that has limited crystal growth in the *a* direction. When comparing the two side view cross sections (Figure 3D,E), we noticed the arms in the same column are angled in opposite directions, revealing the two segments have inverted symmetry along the *a* direction with the proposed geometry shown in the molecular representation in Figure 3A. Combining these observations in cryo-TEM 3D reconstructions, we conclude that the ordering of the H-Ndc_10_-Nte_10_ nanofibrils was significantly enhanced in the presence of urea, and that the fibrils consist of a bilayer with inverted symmetry.

**Figure 4:**
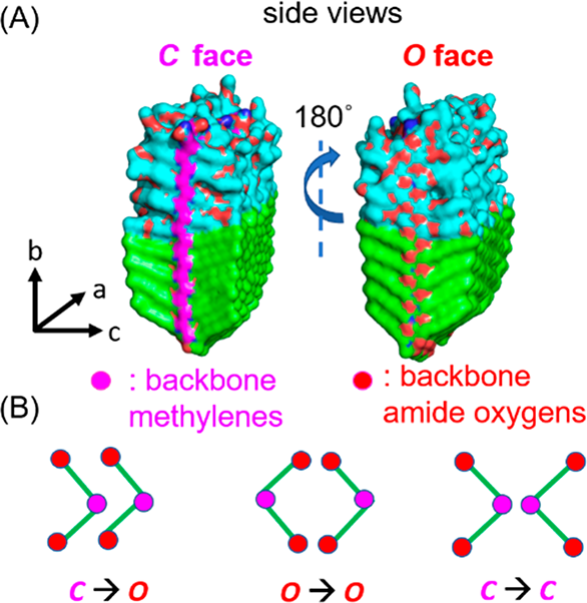
(A) Molecular representations of the two exposed faces of one crystalline stack (C-face and O-face) are different. (B) Three possible bilayer interfaces at the center of the fiber.

The bilayer packing observed in the fibers is not observed in the nanosheets, which have much higher long-range order in the *a* direction. One possible reason is the increased exposure of the hydrophobic *bc* face to the solvent in the fibers. Interestingly, if we look at both sides of the exposed the *bc* surfaces (Figure 4), both contain similar side chains, but one side of the stack displays of an array of backbone amide oxygen atoms along the *a* direction (referred to as the O-face) and should thus be considerably more hydrophilic than the other face which displays an array of methylene groups (referred to as the C-face). Thus, a bilayer could in principle assemble in three possible ways at the internal bilayer interface: O–O, C–O, or C–C (Figure 4B). The C–C is likely to be the most energetically favored, with the hydrophilic O-face exposed to water and the hydrophobic C-face embedded inside of the crystal lattice.

### MD Simulation of the Nanofibrils

To unveil the morphology with full atomic detail, we built an MD model for the bilayer structure in 4 M urea (sample **2**). This system was chosen because of its more ordered crystal structure in the cryo-TEM 3D reconstruction. The nanofibril model is similar to the nanosheet model but has a finite width of only 12 peptoid molecules in the *a* direction. At room temperature, the nanofiber structure was maintained during the entire 90 ns simulation. The initial and relaxed structures can be found in Figure S10. Several key aspects of the 3D reconstruction were in good agreement with the relaxed MD model (Figure 3B–E). At the interface of the two stacks in the *c* direction, the alkane lattice closely matched the spacings and the tilt angle observed in the TEM experiment (Figure 3B). To estimate the tilt angle, backbone *N-N* distances were analyzed. This tilt angle was found at ∼ 71°, which is reasonably close to the experimental tilt angle at 77°, which was determined at the tips of the side chains in Figure 3B. From the top view (*ac* plane in Figure 3C), the decyl side chain adopts the characteristic V-shape within each stack. Interchain distance along the *a* direction was measured by calculating all N–N distance between adjacent chains. The distance was found to be 4.8 ± 0.4 Å, which is in good agreement with the TEM measurements (Figure 3C). From the side view, the distance between alternate decyl side chains from one strand is 5.6 ± 0.1 Å, consistent with the 5.6 ± 0.1 Å from TEM (Figure 3D), and is the characteristic spacing of the all-*cis*-sigma strand secondary structure. Within each segment, the stacks are antiparallel to each other based on the angle from the emanating side chain to the backbone (θ in Figure 2A). The two segments have an inverted symmetry with respect to each other (Figure 3C–E). These quantitative matches for the tilted Ndc lattice are evident that the MD model is capable to reproduce the main features of the experimental model.

**Figure 5:**
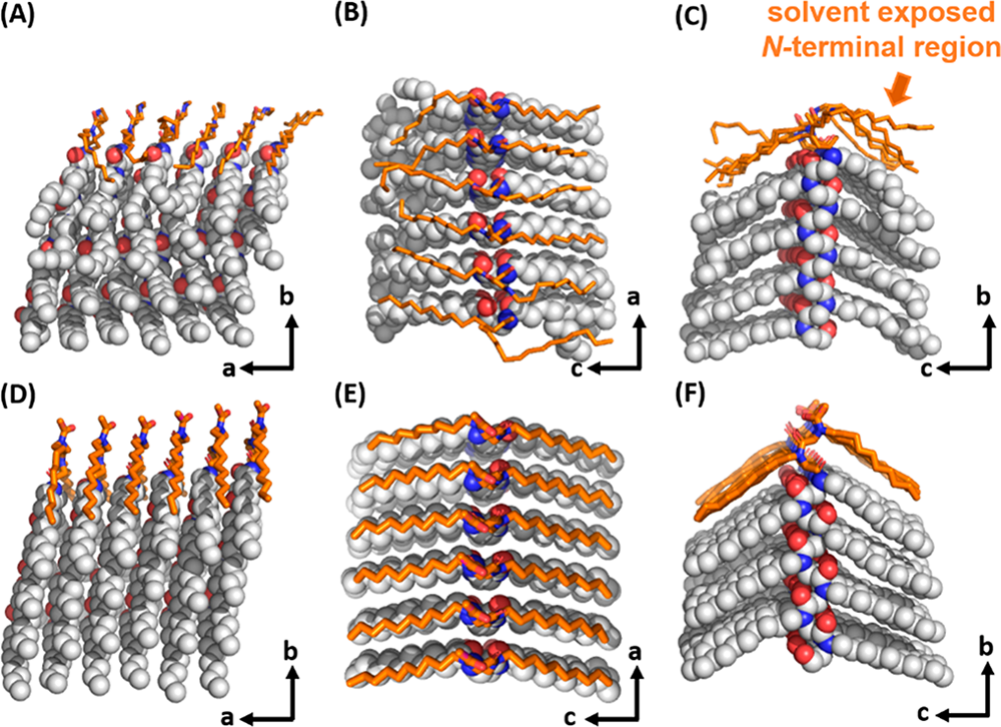
Stacks of lattice from the relaxed MD simulation models of (A–C) H-Ndc_10_-Nte_10_ nanofibrils and (D–F) Ac-Ndc_9_-Nte_9_ nanosheets.^17^ The solvent-exposed Ndc residues neighboring to the N-termini are marked in orange. The computational models evidenced that the solvent-exposed N-terminus destabilized the lattice.

The MD simulations provide key insights into why growth of the peptoid lattice is limited in the *a* direction, and how exogenous small molecules like urea can stabilize the peptoid lattice. In addition, the MD trajectory also reveals structural insights into the disordered regions that are invisible to the TEM 3D reconstruction, such as the polymer–solvent interface and regions of heterogeneity. Considering the interactions in the *a* direction, two phenomena appear to play a significant role. First, we consider the role of the amorphous Nte domains. While the amorphous Nte blocks extend into the solvent, their disordered side chains are observed to partially interact with the solvent exposed Ndc surface as shown in Figure 3B,C, which may sterically hinder additional polymer chains from approaching and attaching to the nanocrystal. Second, less-ordered Ndc monomers are clearly evident (orange regions in Figure 5A–C) near the solvent-exposed N-terminal region of the nanofibril lattice. The N-terminal Ndc side chains are less extended and unable to align with the underlying lattice. In contrast, the acetyl-capped nanosheets from our prior research^17^ were found to have highly ordered N-terminal Ndc residues (Figure 5D–F). Relatedly, the N-terminal portion of the backbone on the outer edges of a molecular stack tend to slightly peel off from their inner molecular neighbors (see also Figure S12). This peeling off indicates a less-ordered lattice near the exposed Ndc surface and at the N-termini, which likely hinder the growth of nanofiber in width (along the *a*-axis).

**Figure 6:**
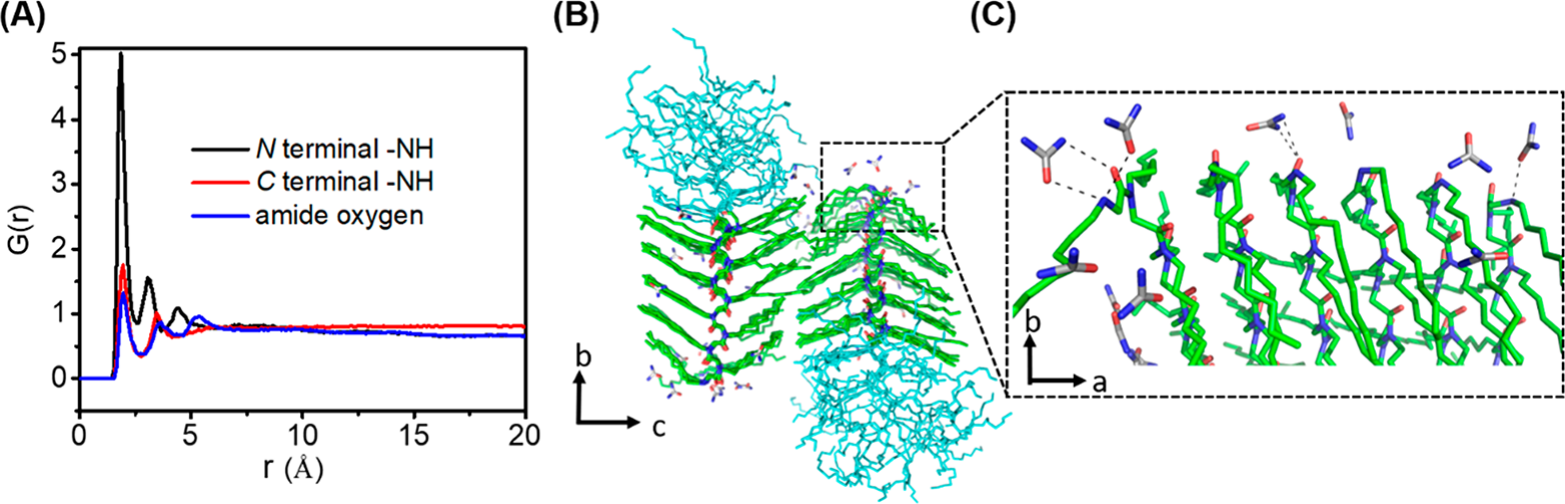
Interaction of urea with the peptoid lattice. (A) Radial distribution functions of urea–peptoid lattice: N-terminal nitrogen to urea oxygen (black), C-terminal −NH to urea oxygen (red), N-terminal amide oxygen to urea hydrogen (blue). (B) Snapshot from the relaxed MD simulation model showing that urea locates at the surface of the Ndc lattice; The N-terminal region highlighted in (B) is shown in (C), which shows urea forms hydrogen bonds (black dashed lines) with the solvent-exposed N-termini.

In order to explore the role of urea in stabilizing the peptoid lattice, we first examined the proximity of urea to various regions of the peptoid lattice. The molar ratio of urea with respect to water was calculated. It was found to be 0.31 within 2.5 Å of the Ndc blocks, and 0.20 within 2.5 Å of the Nte blocks. Since 4 M urea solution has a molecular ratio of 0.072, we can conclude that urea is more concentrated at the peptoid surface, especially near the Ndc blocks. Its presence at the peptoid–solvent interface could possibly stabilize the nanostructure. To further quantify the degree of association of urea with the peptoid assembly surface, radial distribution functions (RDFs) were extracted for various pairs of atom types: the N-terminus, the C-terminus, and the backbone (Figure 6A). Figure 6A shows the interactions at the N-terminus is 5 times more intense than the other two and bulk solvents. The urea molecules within 2.5 Å of the Ndc blocks were depicted for two neighboring stacks in a representative snapshot of the simulation (Figure 6B). Many urea molecules are observed to interact the peptoid N-termini via both its oxygen and its nitrogen atoms (Figure 6C). The high concentration of urea in this region suggests that urea might further stabilize the nanostructure via a network of hydrogen bonding interactions with the N-termini, promoting the formation of a more ordered lattice in the *a* direction. Additionally, DFT calculations in a model system also point to atom-level interactions by which urea may order charged peptoid N-termini (Figure S20). In the *c* direction, the “tip-to-tip” interaction appears to be less impacted by changes at the N-termini, since fibril lengths of greater than 200 nm are observed. However, some deviations in lattice registration are observed along the *c* direction, resulting in some rotation about the *c*-axis (Figure S10).

### Further Stabilization of the N-Terminus

**Figure 7:**
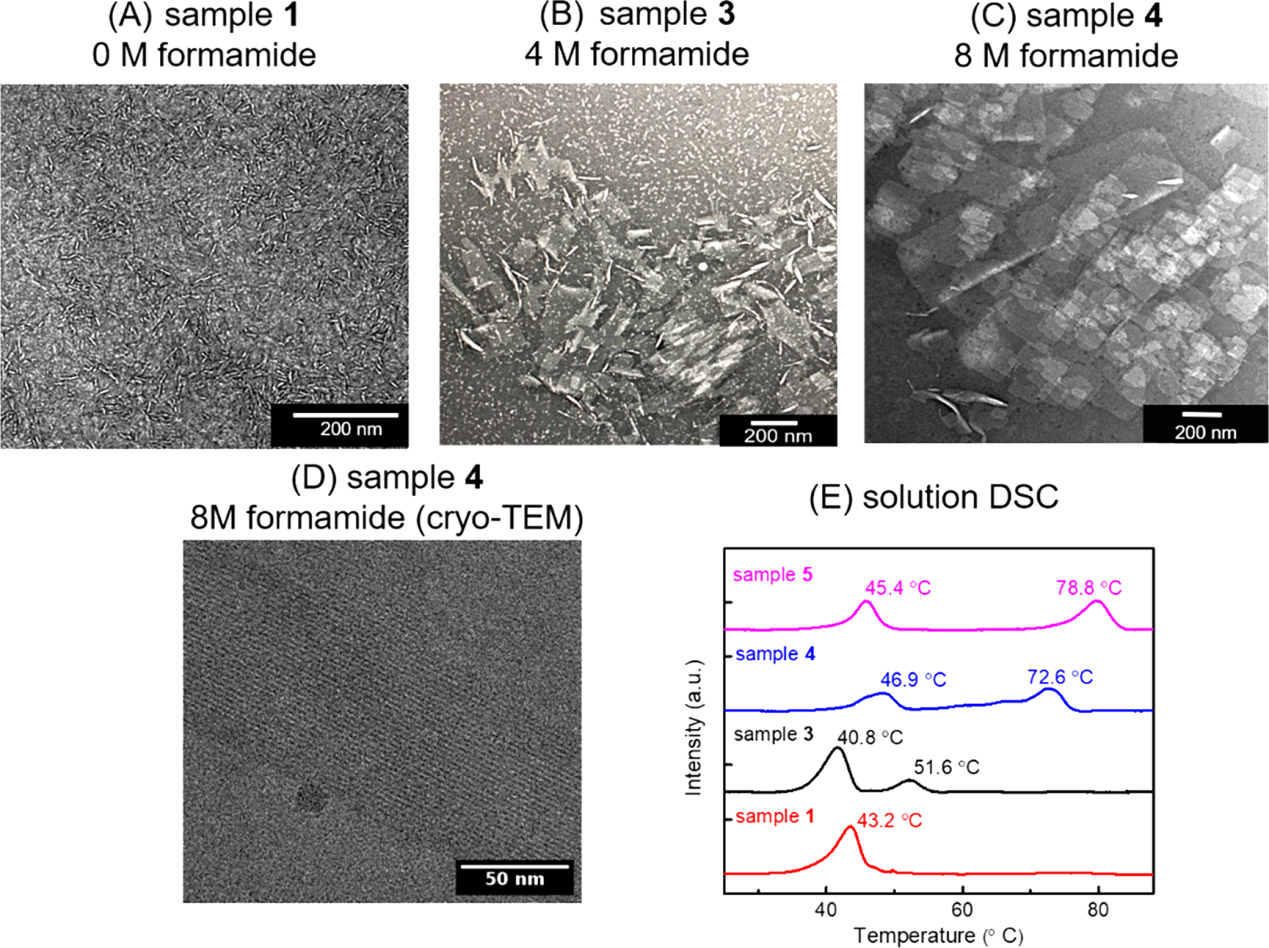
TEM characterizations on the H-Ndc_10_-Nte_10_ assemblies in: (A) pure water (sample **1**), (B) 4 M formamide (sample **3**), or (C) 8 M formamide aqueous solutions (sample **4**). (D) High-resolution cryo-TEM image of sample **4**. (E) Solution DSC analysis on samples **1**, **3**, **4**, and **5**. Images in A–C were obtained from negative stained dry samples. Image in D was obtained from unstained frozen hydrated sample.

Since urea is capable of improving the molecular ordering of the crystalline lattice, we next attempted to push this effect even further by exploring higher concentrations of urea. Due to the solubility limitation of urea in THF, we used formamide, a structurally similar compound, with a higher solubility in THF/water. H-Ndc_10_-Nte_10_ was allowed to assemble by evaporating THF from water under three different conditions: no formamide (sample **1**), with 4 M (sample **3**) and with 8 M formamide (sample **4**) in Figure 7. Interestingly, we observe a dramatic change in the aspect ratio from 1D to 2D nanostructures with increasing formamide concentration. TEM images revealed that nanofibrils were formed in the absence of formamide as expected (sample **1**, Figure 7A), and a mixture of fibrils with small sheets were formed with 4 M formamide (sample **3**, Figure 7B), and large nanosheets were formed in 8 M formamide (sample **4**, Figure 7C). The thickness of sample **4** (4.4 ± 0.2 nm, Figure S7B) is comparable to that of sample **5** (4.9 ± 0.2 nm, Figure S7A) as determined by atomic force microscopy (AFM). Cryo-TEM image of frozen hydrated 8 M formamide nanosheet (sample **4,**Figure 7C) reveals long-range order in the *a* direction (>200 nm, Figure 7D), as compared to the fibrils in urea (sample **2**, Figure 1C) which is limited to < 6 nm. Meanwhile, the XRD and solution wide-angle X-ray scattering (WAXS) data (Figures S4 and S5) show the samples with (sample **3** and **4**) and without (sample **1**) formamide share the same crystal lattice spacings. However, there is a distinct sharpening of both the *c* (001) and *a* (102) peaks (fwhm) in the presence of formamide: fwhm (001) and fwhm (102) in sample **4** decreased 43 and 39%, respectively, with formamide (Table S2). This is further evidence that the nanofiber and the nanosheet have nearly identical crystal lattices, and that formamide can increase local ordering, which in turn results in enhanced crystal growth in the *a* direction. Meanwhile, in the solution DSC analysis in Figure 7E, a significant new second phase transition temperature (*T*_2_) appears in the formamide assemblies, which is a characteristic feature of *N*-alkyl glycine nanosheets.^19^ The value of *T*_2_ increases from 51.6 °C in 4 M formamide (sample **3** in Figure 7E) up to 72.6 °C in 8 M formamide (sample **4** in Figure 7E), which is comparable to the *T*_2_ of F-Ndc_10_-Nte_10_ nanosheets (sample **5** in Figure 7E). Thus, formamide can change the nanoscale morphology from fiber to sheet by increasing the local molecular ordering at the N-terminus. To investigate if the effect of formamide is reversible, we dialyzed the assembled nanostructures against water. We observed the formamide nanosheets (sample **4**, Figure S7C) dissociated to a mixture of nanofibrils and smaller nanosheets upon the removal of formamide by dialysis (Figure S7D), whereas the formyl end-capped nanosheets (sample **5**, Figure S7A) remained intact upon dialysis (Figure S7B). This suggests that weak, noncovalent interactions at the N-terminus can reversibly dictate the nanoscale morphology.

To further understand the role of N-terminus in the assembly, we flipped the order of the two blocks in H-Ndc_10_-Nte_10_ to create the reverse sequence (H-Nte_10_-Ndc_10_). In this reverse design, the crystalline Ndc block is further away from the N-terminus as compared to the original H-Ndc_10_-Nte_10_ sequence. The N-terminal portion of the Ndc block has very different chemical environments: H-Ndc_10_-Nte_10_ has solvent-exposed secondary amine termini, and H-Nte_10_-Ndc_10_ is acylated by the entire Nte domain at the N-terminus. TEM images show the reverse sequence assembled into large nanosheets in water (sample **6**, Figure S7) rather than into nanofibrils as the original sample does (sample **1**, Figure 1C). Furthermore, solution DSC analysis of the reverse sample (sample **6**, Table 1) exhibited two thermal transition peaks, similar to the N-terminal formyl-capped nanosheets (F-Ndc_10_-Nte_10,_ sample **5**, Figure 7E), whereas only a single transition peak was observed in the original sample with a free N-terminus (H-Ndc_10_-Nte_10_, sample **1**, Figure 7E). Taken together, these data suggest that sample **6** has a more organized Ndc crystalline domain and thus is capable of forming nanosheets with long-range order in the *a* direction.

## Conclusions

We identified the crucial morphology directing role of the N-terminus in the assembly and crystallization of Ndc_10_-Nte_10_ diblock co-polypeptoids in aqueous solution. We observed acylation at the N-terminus resulted in a distinct morphological change from less-ordered nanofibrils to well-ordered nanosheets, as evidenced by cryo-TEM 3D reconstruction, solution DSC, XRD, and MD simulation. We also demonstrated previously proposed nanotubes^18^ are rather nanofibrils, with great similarity to previously reported crystalline nanosheets, sharing a rectangular lattice observed in many peptoid nanostructures.^68,69−10,14,15,17,19,37^ Even though the nanofibrils are not as highly ordered as the nanosheets, the ability to image them at multiple angles allowed direct observation of the side view of a peptoid chain in the plane of the backbone (*bc* plane, Figure 3D,E), which is difficult to achieve with a large 2D nanosheet. Importantly, the all-*cis*-sigma strand conformation hypothesized for many years^15^ could be directly observed by high-resolution cryo-TEM. Although there has been much indirect evidence from XRD,^9,19^ solid-state NMR,^57^ and MD simulation,^9,14,17,70^ the repeating backbone secondary structure has never been directly observed in real space. We further demonstrated long-range ordering along the *a* direction in the crystal lattice can also be significantly enhanced by exogenous small molecules (e.g., urea, formamide). Simulations suggest hydrogen bonding between urea/formamide and the solvent-exposed free N-terminal region significantly stabilizes the ordering of the entire crystal lattice, and thus enhances the crystal growth into nanosheets with long-range order in two dimensions. When we consider the extent of growth in the *a* direction, fibrils are limited to a length of 5–6 nm, whereas in nanosheets, the *a* direction can be up to microns in length. This extent of growth is interesting because interactions in the *a* direction are known to be stronger than in the *c* direction in acylated *N*-alkyl glycine lattices (as evidenced by temperature-dependent SAXS/WAXS and calorimetry studies),^19^ and that the nanosheets typically grow longer in the *a* direction than the *c* direction,^37^ which is opposite to that observed in the nanofibril (Figure S9). Taken together, our results illustrate how both intra- and intermolecular interactions at the N-terminal nitrogen atoms dramatically impact the degree of the long-range order as well as the structural homogeneity of the peptoid lattice in the *a* direction. This understanding of the importance of the chain termini in peptoid crystal lattices will greatly accelerate the design of precisely ordered, functional biomimetic nanostructures.

## Experimental Section

### Materials

2-(2-(2-Methoxyethoxy)ethoxy)ethylamine was purchased from Aurum Pharmatech LLC (98% purity), and *n*-decyl amine was purchased from TCI (>98% purity). *N*,*N*′-Disopropylcarbodiimide (DIC) was purchased from Chem-Impex International, Inc. (≥99.5% purity). 4-Methylpiperidine was purchased from Beantown Chemical (98% purity). Bromoacetic acid was purchased from ACROS Organics (≥98% purity). Formic acid (≥99% purity) was purchased from ThermoFisher Scientific Inc. Rink amide MBHA resin (0.64 mmol/g) was purchased from Protein Technologies, Inc. *N*,*N*′-Dimethylformamide (DMF) was purchased from OmniSolv Inc. Trifluoroacetic acid (99% purity) and *N*-methylpyrrolidinone (NMP) (99% purity) were purchased from Sigma-Aldrich. All other needed reagents, 1,2-dichloromethane (DCM), acetonitrile (ACN), and isopropyl alcohol (IPA), were all purchased from VWR Chemicals. All chemicals and solvents were used without further purification.

### Synthesis of Co-polypeptoids

All Ndc_9_-Nte_9_, Ndc_10_-Nte_10_, and Nte_10_-Ndc_10_ diblock co-polypeptoids were synthesized by solid-phase submonomer synthesis using a Symphony X peptide synthesizer at a scale of 100 mg Rink amide resin (0.64 mmol/g) by adapting reported procedures.^7^ All resins were first swelled in DMF for 10 min followed by N-terminal FMOC deprotection using 20 vol % of 4-methylpiperidine in DMF. The addition of peptoid to each monomer consisted of a two-step monomer addition cycle. A bromoacylation was first performed with bromoacetic acid (0.8 M) and DIC (0.8 M) in DMF for 20 min at room temperature. Next, a displacement reaction was performed by adding submonomer amine (1 M in DMF) for 30 min at room temperature. The bromoacylation and displacement cycle was repeated for each peptoid monomer in the target sequence from C-terminus to the N-terminus. All chemical steps were followed by washing to get rid of the unreacted reagents.

Formylation at the N-terminus of the full-length peptoids was performed on the resins in 6 mL PP disposable, fritted reaction vessels. One hundred milligrams of the peptoid-grafted resins was swelled in 2 mL of DMF for 20 min before draining the solvent. A 1 M solution of DIC in NMP was added (1 mL) followed by the addition of 1 M formic acid in NMP (1 mL). The reaction mixture was shaken for 20 min at room temperature before draining. The reaction was repeated once before being washed with DMF (3 × 2 mL) and DCM (3 × 2 mL) to remove the unreacted reagents.

### Cleavage, Purification, and Characterization of the Co-polypeptoids

The crude peptoids were cleaved from the resin by suspending the resins in a trifluoroacetic acid (TFA) solution (95% v/v) in water (6 mL), and shaken for 20 min at room temperature. The cleavage solution was filtered and washed with DCM (3 × 4 mL), and the volatiles were evaporated using a Biotage V-10 evaporator to yield a faint yellow gel (∼220 mg, yield = 85.7%).

The crude peptoids were purified by reverse-phase high-performance liquid chromatography (HPLC). The freshly cleaved peptoids were dissolved in ACN/IPA/water (60/10/30% v/v) (6 mL). The peptoid solution was first sonicated at room temperature for 10 min and then centrifuged at 13.2 rpm for 3 min to remove any particulates and possible aggregates. The clear supernatant was loaded onto the Waters reverse-phase HPLC with a XSelect HSS cyano column (5 μm, 18 × 150 mm^2^): solvent A (10% IPA in water containing 0.1% TFA) and solvent B (10% IPA in ACN containing 0.1% TFA). A flow rate of 12 mL/min was used, with a linear gradient at 60–95% B over 30 min. The fractions were analyzed by a reverse-phase liquid chromatography mass spectrometer equipped with an analytical XSelect HSS cyano column (5 μm, 4.6 × 150 mm column) and a MicroTOF electrospray mass spectrometry. Solvent A is 10% IPA in water containing 0.1% TFA), and solvent B is 10% IPA in ACN containing 0.1% TFA. Solvent A is ultrapure water with 10 vol % of IPA and 0.1 vol % of TFA, and solvent B is ACN with 10 vol % of IPA and 0.1 vol % of TFA. The flow rate is 0.4 mL/min with a linear gradient at 60–95% solvent B over 30 min. The HPLC fractions with pure compound were collected by lyophilizing from acetonitrile/water (1:1, v/v) using a Genevac evaporator to yield a fluffy white powder with > 99% molecular purity.

### Self-Assembly of the Co-polypeptoids

Purified peptoids were dissolved in THF at 4 mg/mL, followed by the addition of an equal volume of ultrapure water to obtain an assembly solution containing peptoid at 2 mg/mL in THF/water (1/1, v/v). This is close to the solubility limit of the peptoids in THF/water in order to maximize the assembly rate. The assembly vials were capped loosely and the THF was allowed to evaporate slowly at 4 °C for up to 14 days. The solutions which retained ∼ 5% residual THF were used directly in solution DSC, solution WAXS and directly diluted ∼100 times for cryo-TEM analysis.

### Differential Scanning Calorimetry Analysis on Peptoid-Assembled Nanostructures in Solution and in the Dry State

The thermal behavior of the assembled peptoid nanostructures were analyzed in solution by nanodifferential scanning calorimeter (CSC Model 6100, TA instrument). The assembly products were analyzed in the aqueous solutions (4 mg/mL) and the solution background were degassed with a degassing station (model number of 6326, TA instrument) at 80 mbar for 20 min. 650 μL of the sample and background solution were loaded into the sample and reference capillary respectively. The solutions were equilibrated at 15°C for 15 min following by heating at 1 °C /min at temperature range from 15 to 110 °C under a pressure of 3.0 atm. The data were further exported and analyzed by Origin software.

DSC analysis on dry peptoid assembled nanostructures was performed on a TA Q200 differential scanning calorimeter. Peptoid nanosheets/nanofibrils aqueous solutions (4 mg/mL) were pipetted into the preweighted aluminum T zero pans and dried under vacuum, and the process was repeated for several times until enough sample (∼1–2 mg) was placed. The pans were sealed with aluminum T zero lids, and an unloaded pan with lid of the same type was used as reference for all the measurements. Each sample was quickly quenched to 0 °C and then heated to 120 °C at a rate of 10 °C/min.

### AFM Characterization

Ex situ (in air) AFM imaging of the peptoid assembled nanostructures were performed on an Asylum MFP-3D (Oxford Instruments) atomic force microscope in tapping mode. The peptoid assembly solution was diluted for 10 times (0.4 mg/mL) with ultrapure water. Five microliters of the diluted solution was loaded onto precut 4 in. silicon wafer (4 in. wafer with 5 × 7 mm chips, Ted Pella, Inc.), which was plasma cleaned in a Harrick plasma cleaner using a mix of Ar/O_2_ (25/75 v/v). The drop was then quickly dried with a stream of nitrogen. TAP 150 AL-G tips were used with resonant frequency at 150 kHz and the force constant at 5 N/m. The AFM images were processed and analyzed using Gwyddion software.

### Powder X-Ray Diffraction

One hundred microliters of peptoid nanofibrils/nanosheets aqueous solutions (4 mg/mL) was centrifuged in a microcentrifuge tube at 13.2 rpm for 30 min. The peptoid nanostructures were pelleted, and the supernatants were decanted. The nanostructures were washed with ultrapure water, redissolved, vortexed, and recentrifuged three times and washed with ultrapure water (100 μL) to remove any residual free urea or formamide. The nanostructures were further dried with a stream of nitrogen.

The dried peptoid nanostructures were redissolved in 1 μL of Milli-Q water in the Eppendorf tubes, and the concentrated solutions were pipetted on the MiTeGen micromeshes, which were further dried under house vacuum. The process was repeated for 3 times to provide enough material for analysis.

Powder XRD data of the nanostructures were collected at ALS beamline 8.3.1, using multiple wavelength anomalous diffraction and monochromatic macromolecular crystallography. Beamline has a superbend source with an energy range of 5 to 17 keV. The sample was collected at 1.158Å X-ray beam. The sample-to-detector distance is 500 nm. The data were analyzed using Fit2D software.

### Solution WAXS Analysis of the Nanofibrils/Nanosheets

Solution WAXS data were collected at ALS beamline 7.3.3 in Lawrence Berkeley National Lab. 60 μL of the nanosheet/nanofibril assembly product (4 mg/mL) were loaded into the quartz capillary (O.D. = 1.5 mm). The sample was collected at 10 keV (1.24 Å) X-ray beam, and the sample-to-detector distance is 286.65 nm. The data were analyzed using Origin software.

### Cryo-TEM Data Collection

A 3 μL droplet of nanostructures in aqueous suspension was applied to the AuFlat grid (Protochips Inc.), which has a holey gold/palladium alloy film on a gold grid. The grid was blotted by filter paper and then plunged into liquid ethane to obtain vitrified specimens using a Vitrobot (FEI Company). The vitrified specimens were imaged with a FEI Krios (FEI Inc.) operated at 300 kV with a K3 direct detection camera and postcolumn energy filter (Gatan Inc. slit width at 20 eV) and JEOL-3200FSC (JEOL Inc. Japan) equipped with a K2 Summit direct detection camera and in-column energy filter (width at 25 eV). Dose-fractionation movies were recorded with the accumulated dose about 20 e/Å^2^. Pixel size is 0.7 Å (referred to the image), and the defocus value was set to −1 μm during the low dose data collection using serialEM.^58^

### Single-Particle 3D reconstruction

Dose–fractionation movies were aligned and summed using Motioncorr2^59^ to obtain dose-weighted images. These images were imported into CryoSparc^50^ for contrast transfer function (CTF) estimation. About 600 small sections (256 by 256 pixels) along the fibers were manually picked up. These sections were sorted and averaged using 2D classification to generate initial templates for the automated pickup using filament tracer. More sections extracted by the first-round automated pickup were used for 2D classification to generate more accurate initial templates. After two rounds of automated pickup using initial templates generated by 2D classification, all sections were sorted and averaged to rule out the sections without fiber structures. The 2D classifications results obtained from H-Ndc_10_-Nte_10_ nanofibrils in water (sample **1**) and H-Ndc_10_-Nte_10_ nanofibers in urea (sample **2**) are shown in Figures S9 and S10. Different box sizes were also tested to find the best extraction parameters as shown in Figures S15 and S16. Ab initio 3D reconstruction of initial 3D map was carried out using CryoSparc.^50^ 3D classification was carried out to reveal whether or not the structure is homogeneous.^60^ Only one ordered structure was found as shown in Figure S17. A solvent mask with soft edges was generated by thresholding the contrast in initial map to exclude the water. Local refinement was then carried out to refine the alignment of all sections to the initial model. Local and global CTF were refined using the 3D map generated by local refinement. The spatial resolutions of the final 3D maps of H-Ndc_10_-Nte_10_ nanofibers in water and H-Ndc_10_-Nte_10_ nanofibers in urea are 3.7 and 3.6 Å, respectively, as shown in Figures S13 and S14, according to Fourier shell correlation (FSC) 0.143 criterion (Figure S18).

### Negative Stain TEM Data Collection

A 3 μL droplet of nanostructures/uranyl acetate mixture solution was applied to the copper grid with continuous carbon film (Ted Pella, Inc.) The droplet was blotted using a filter paper from the edge of the grid. Images were obtained from a grid with dried nanostructures using the JEOL-1400Flash (JEOL, Inc. Japan) with a Gatan Oneview camera at room temperature.

### MD Simulation

A CGenFF-based peptoid force field for the peptoid backbone,^61^ and the standard CGenFF for the side chain ligands were employed in this study.^62^ For the protonated N-terminus and the counterion trifluoroacetic acid, the bonded and van der Waals parameters were obtained from the automatic matching tool,^63,64^ and the partial charges were obtained from density functional theory calculations and the RESP algorithm, using TeraChem 1.93.^65,66^ GROMACS 2019.2 was used to perform the molecular dynamics simulations.^67^ With our best intuition from the cryo-TEM 3D reconstruction data, the initial structure was preassembled with H-Ndc_10_-Nte_10_ in the all-*cis*-sigma strand conformation. Peptoid chains were arranged into two segments with the C-face pointing to the center, as shown in Figure 4B. Each segment consists of 6 stacks, and each stack contains 6 molecules. The chirality and direction of the stacks were carefully chosen to achieve the best contact of the Ndc side chains, and the Nte blocks were arranged to protrude into the bulk solvent (Figure S10). Our simulation started with the minimization that included the urea molecules. Ten ns of Langevin dynamics were performed with the Ndc blocks being fixed. This preliminary run resulted in amorphous Nte blocks.^56^ The structure was subsequently solvated by TIP3P water, and the equilibration was carried out in the NpT ensemble at 300K and 1 atm for 90 ns. Several runs of 50 ns were consecutively performed using the same configuration. The potential energy was monitored until its average value did not further decrease, and the trajectory was collected for analysis. For all the steps above, the time step was set to 2 fs and the bond lengths involving hydrogen atoms were fixed using the LINCS algorithm.

## Supporting Information Available

LC-MS characterization of the peptoids; XRD, solution WAXS, AFM, negative-stained TEM, cryo-TEM, and DSC characterization of the assembled nanostructures; DFT calculations and additional MD simulation results (PDF)
