# Supplementary material for: Structural Elucidation of a Polypeptoid Chain in a Crystalline Lattice Reveals Key Morphology-Directing Role of the N-Terminus
Source: ACS Nano. 2023 Feb 23;17(5):4958–70. doi: 10.1021/acsnano.2c12503 (PMC10018772; doi:10.1021/acsnano.2c12503)
Supplement: Supplementary file 1 — nn2c12503_si_001.pdf [file nn2c12503_si_001.pdf]

Structural Elucidation of a Polypeptoid Chain in a Crystalline Lattice Reveals Key Morphology-Directing Role of the *N*-Terminus

Tianyi Yu,[1,3] Xubo Luo,[3] David Prendergast,[3] Glenn L. Butterfoss,[4] Behzad Rad,[3]

Nitash P. Balsara,[1,2] Ronald N. Zuckermann[3], Xi Jiang[1]**

1. Materials Sciences Division, Lawrence Berkeley National Laboratory, Berkeley, CA 94720, USA

2. Department of Chemical and Biomolecular Engineering, University of California, Berkeley, CA 94720, USA

3. Molecular Foundry, Lawrence Berkeley National Laboratory, Berkeley, CA 94720, USA

4. Center for Genomics and Systems Biology, New York University, Abu Dhabi, United Arab Emirates

*Corresponding author: xijiang@lbl.gov; rnzuckermann@lbl.gov

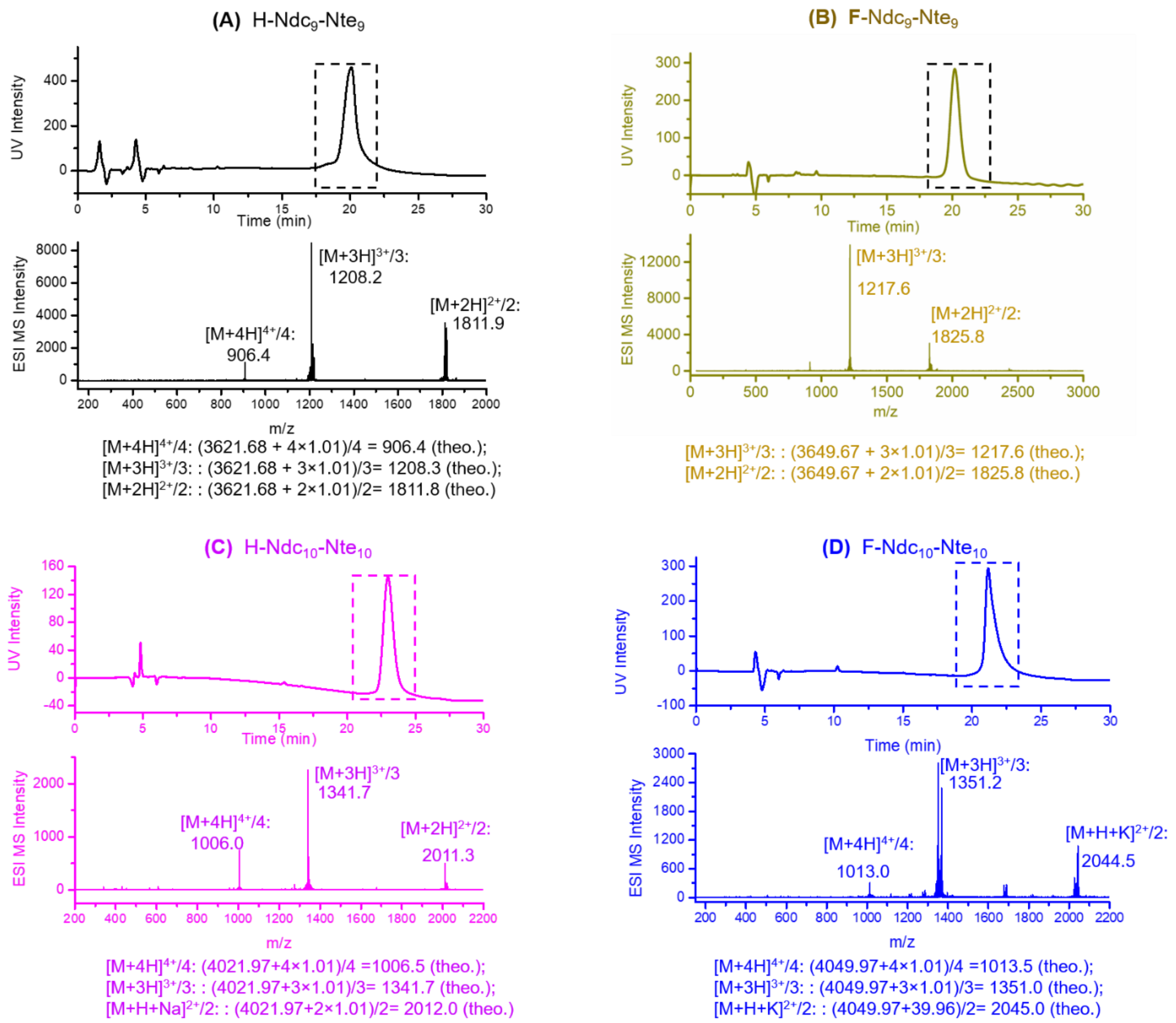

Figure S1. LC-MS trace of purified (A) H-Ndc$_9$-Nte$_9$, (B) F-Ndc$_9$-Nte$_9$, (C) H-Ndc$_{10}$-Nte$_{10}$, and (D) F-Ndc$_{10}$-Nte$_{10}$ with gradient of 60-95% ACN in H_2O in 30 min. Top images are the LC traces and the bottom images are the mass spectra of the selected product peak.

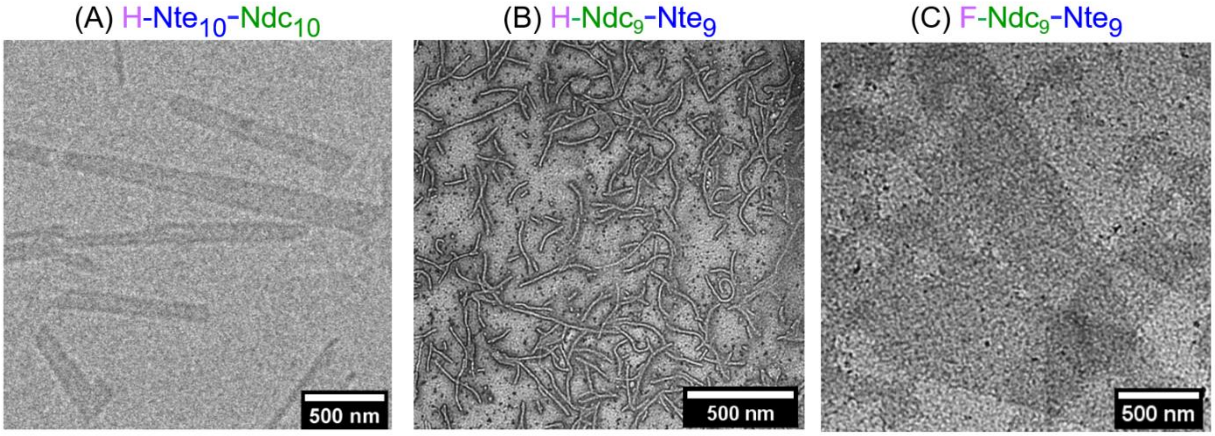

Figure S2. Negative stain TEM characterizations on the (A) H-Nte_{10}-Ndc_{10} nanosheets, (B) H-Ndc_9-Nte_9 nanofibers, and (C) F-Ndc_9-Nte_9 nanosheets.

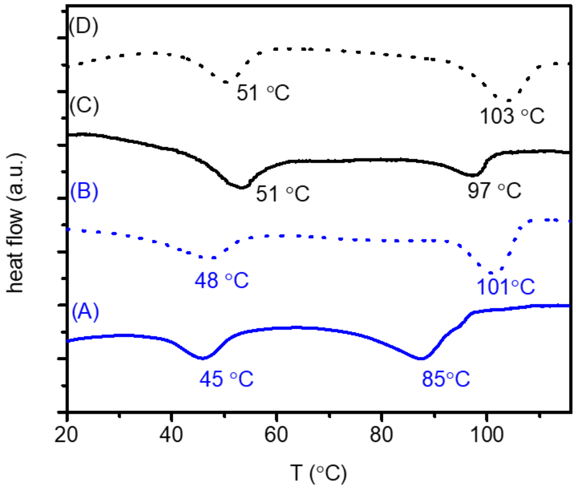

Figure S3. DSC endotherms of the dry samples: (A) H-Ndc_9-Nte_9 nanofibers, (B) F-Ndc_9-Nte_9 nanosheets, (C) H-Ndc_{10}-Nte_{10} nanofibers, and (D) F-Ndc_{10}-Nte_{10} nanosheets.

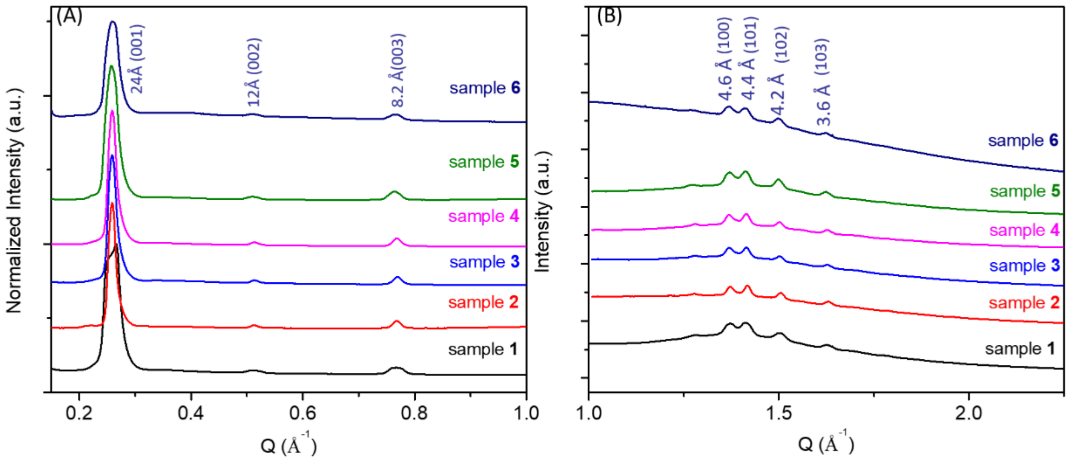

Figure S4. XRD measurements of the dry samples: H-Ndc_{10}-Nte_{10} nanofibers assembled in water (sample **1**), H-Ndc_{10}-Nte_{10} nanofibers assembled in 4M urea aqueous solution (sample **2**), H-Ndc_{10}-Nte_{10} nanofibers/nanosheets assembled in 4M formamide aqueous solution (sample **3**), H-Ndc_{10}-Nte_{10} nanosheets assembled in 8M formamide aqueous solution (sample **4**), F-Ndc_{10}-Nte_{10} nanosheets assembled in water (sample **5**), and H-Nte_{10}-Ndc_{10} nanosheets assembled in water (sample **6**). Figure (A) shows peak (001) at q = 24 Å corresponding to the *c* dimension, and Figure (B) shows peak (100) at q = 4.6 Å corresponding to the *a* dimension.

Table S1. Summary of DSC measurements of the dry nanofibers and nanosheets.

sample	morphology[a.]	T_1[b] (˚C)	T_2[c] (˚C)	ΔH_1[b] (J/g)	ΔH_2[c] (J/g)	ΔH_{total}[d] (J/g)
H-Ndc_9-Nte_9	fiber	45	85	9.1	17.0	26.0
F-Ndc_9-Nte_9	sheet	47	101	18.3	22.9	41.2
H-Ndc_{10}-Nte_{10}	fiber	51	97	13.2	21.0	34.2
F-Ndc_{10}-Nte_{10}	sheet	51	103	14.9	27.7	42.6

[a.] Determined by negative stain TEM. [b.] T_1 and ΔH_1 represent the first thermal transition temperature and the enthalpy change upon heating. [c.] T_2 and ΔH_2 represent the second thermal transition temperature and the enthalpy change upon heating. [d.] Total enthalpy change upon heating (ΔH_{total}) = $\Delta H_1 + \Delta H_2$

Table S2. Summary of XRD measurements of the dry nanofibers and nanosheets.

#	samples	peak (001)[a]		peak (102)[b]	
		FWHM[c.]	r_{001}[d]	FWHM[e.]	r_{102}[f.]
1	H-Ndc_{10}-Nte_{10} in water	0.03	2.0	0.031	1.6
2	H-Ndc_{10}-Nte_{10} in 4M urea	0.015	1.0	0.019	1.0
3	H-Ndc_{10}-Nte_{10} in 4M formamide	0.017	1.1	0.019	1.0
4	H-Ndc_{10}-Nte_{10} in 8M formamide	0.017	1.1	0.019	1.0
5	F-Ndc_{10}-Nte_{10} in water	0.026	1.7	0.023	1.2
6	H-Nte_{10}-Ndc_{10} in water	0.027	1.8	0.024	1.3

[a.] peak (001) at q = 24 Å corresponding to the *c* dimension, [b.] peak (102) at q = 4.2 Å corresponding to the *a* dimension; [c.e.] full width at half maximum (FWHM) of peak (001) and peak (102), respectively; [d. f.] FWHM ratio over sample **2** for peak (001) and peak (102), respectively.

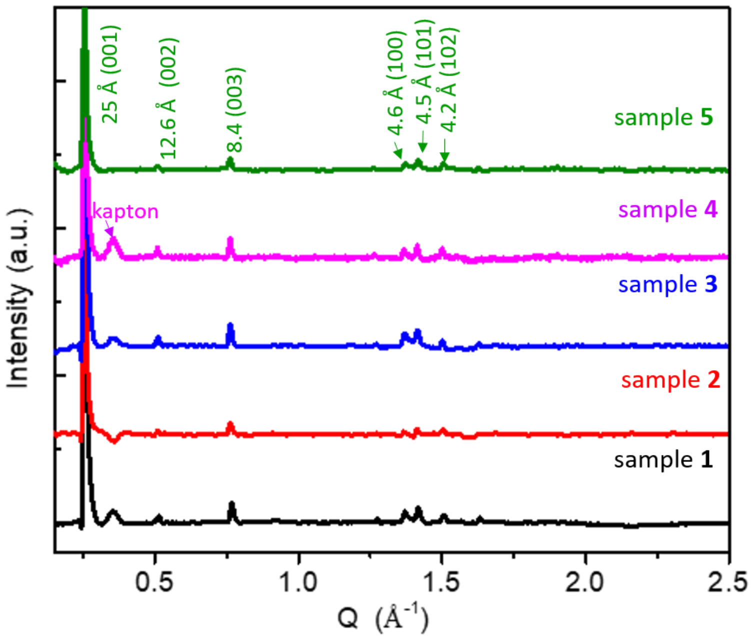

Figure S5. Solution WAXS measurements of the peptoid nanofiber/nanosheets (sample **1-5** in Table S2). Solution WAXS analysis showing peak (001) at q = 24 Å corresponding to the *c* dimension, and peak (100) at q = 4.5 Å corresponding to the *a* dimension. The data shown is after background subtraction.

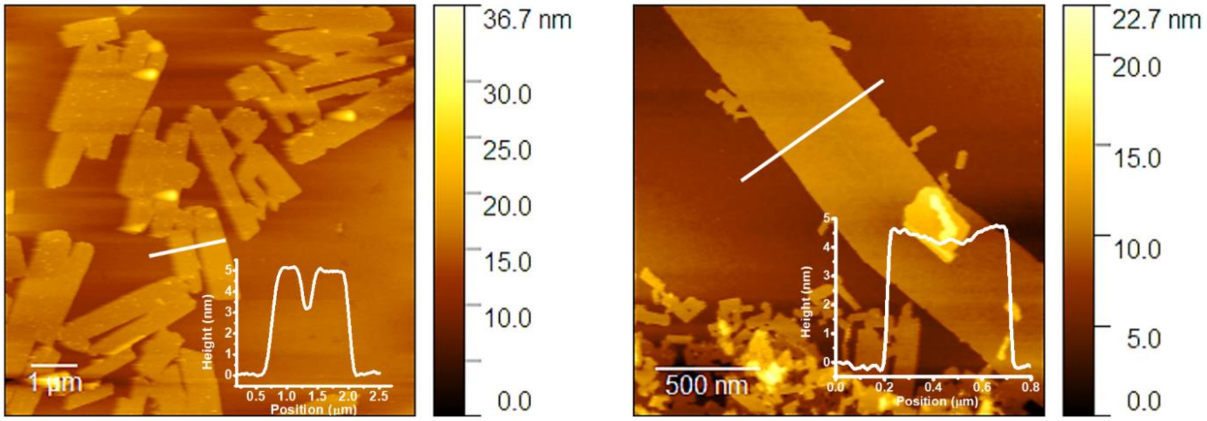

Figure S6. Representative AFM height images of (A) F-Ndc$_{10}$-Nte$_{10}$ (sample **5**) and (B) H-Ndc$_{10}$-Nte$_{10}$+8M formamide nanosheets (sample **4**). The inset graphs are the thickness profiles of the nanosheets. The average thickness of (A) sample **5** (B) sample **4** are 4.9 ± 0.2 nm and 4.4 ± 0.2 nm, respectively.

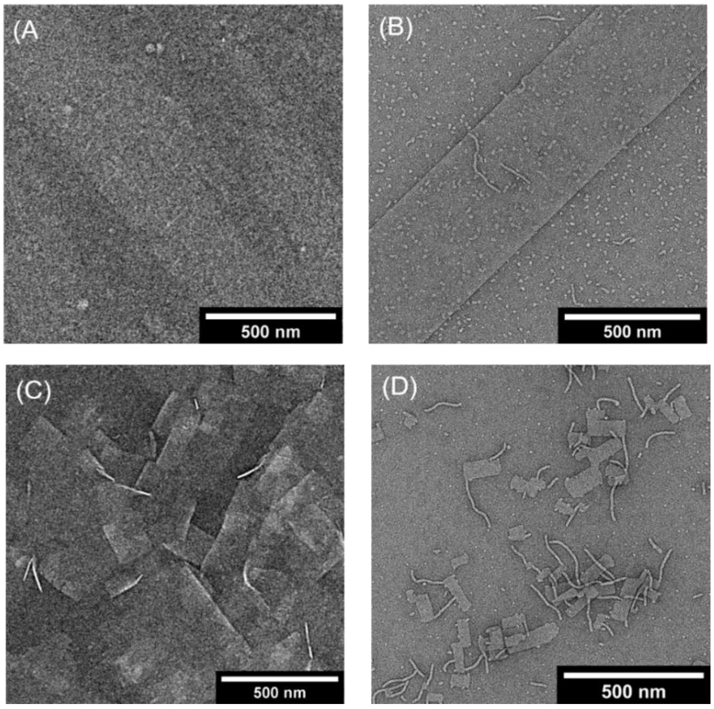

Figure S7. (A)-(B) F-Ndc$_{10}$-Nte$_{10}$ nanosheets (sample **5**) (A) before and (B) after dialysis in MilliQ water at room temperature for 18 hrs; (C)-(D) H-Ndc$_{10}$-Nte$_{10}$+8M formamide nanosheets (sample **4**) (C) before and (D) after dialysis in MilliQ water at room temperature for 18 hrs. The MWCO of the dialysis bag is 500-1000 Da.

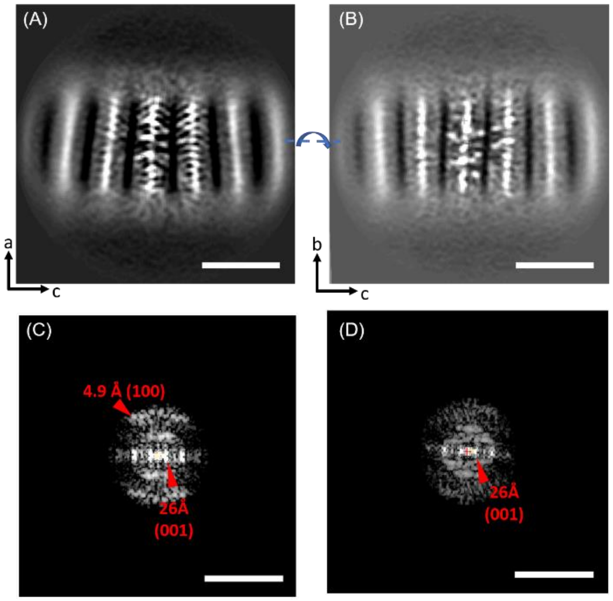

Figure S8. (A)-(B) Averaged TEM slices and (C)-(D) the corresponding FFTs from cryo-TEM 3D reconstruction of H-Ndc_{10}-Nte_{10} nanofibril in water (sample **1**). (A) and (C) show the *ac* plane (top view). (B) and (D) shown the *bc* plane (side view). Slice thickness = 14 Å. Scale bar is 5 nm.

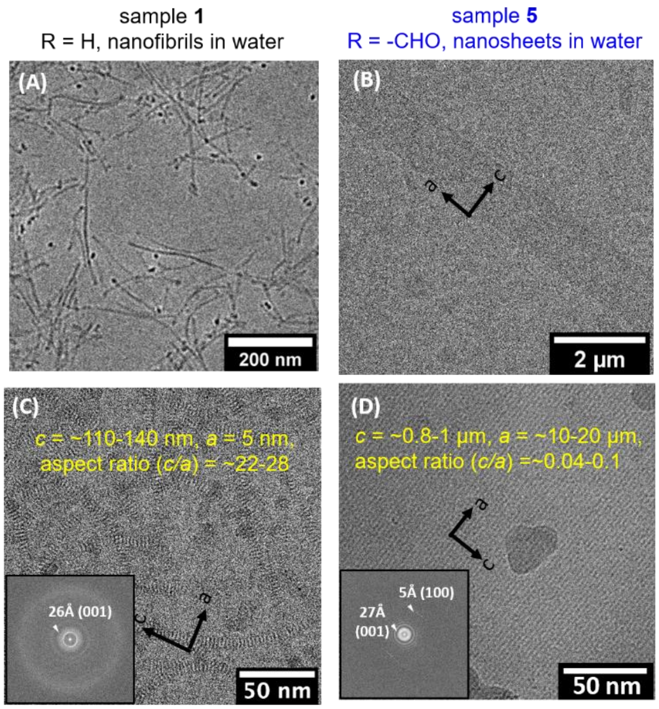

Figure S9. (A)-(B) Low magnification, and (C)-(D) high magnification of sample **1**, and **5**. In the high magnification images (C)-(D), characteristic stripes are observed at d = 26 ± 2 Å. Crystallographic axes are indicated (black arrows). Dark areas in all images represent the electron dense regions. The aspect ratios for the two samples are shown in Figure C and D, respectively. The fast Fourier transforms (FFTs) are shown in the insets in (C)-(D).

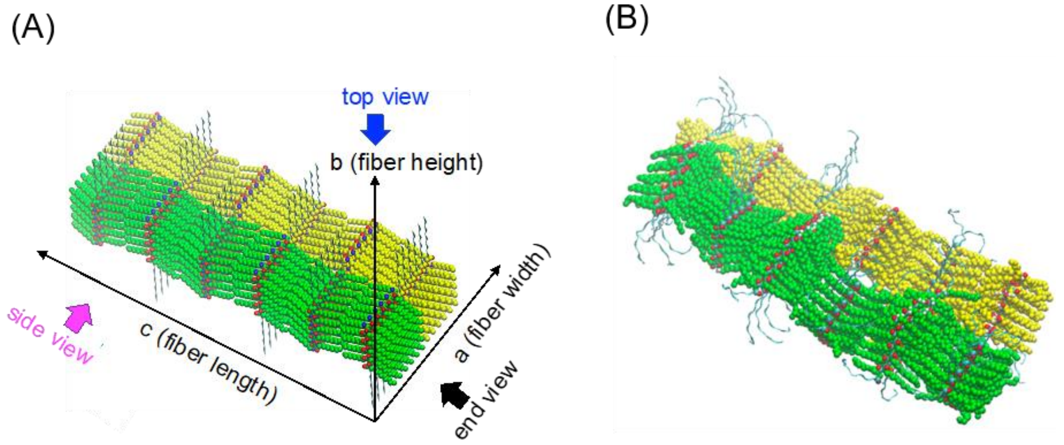

Figure S10. (A) Initial and (B) relaxed computational models for sample **2** using MD simulation. Only the polypeptoid molecules are shown. Two Ndc segments are colored in green and yellow, respectively, while Nte backbones are represented by ribbons. Some deviations in lattice registration are observed along the *c* direction in the relaxed model, resulting in some rotation about the *c*-axis in (B).

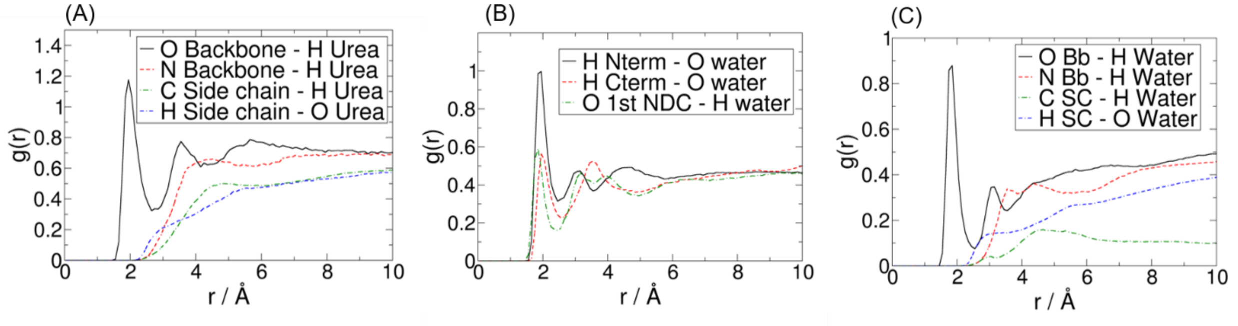

Figure S11. Radial distribution functions of urea – peptoid and water – peptoid. (A) urea to the solvent-exposed Ndc in the *bc* plane. (B) water to *N*-terminus and *C*-terminus; (C) water to the solvent-exposed Ndc in the *bc* plane (bb: backbone, sc: side chain). All the peaks appear to be lower than the pair of oxygen in urea with the -NH at the *N*-terminus in Figure 6A, indicating the strongest correlation of the latter.

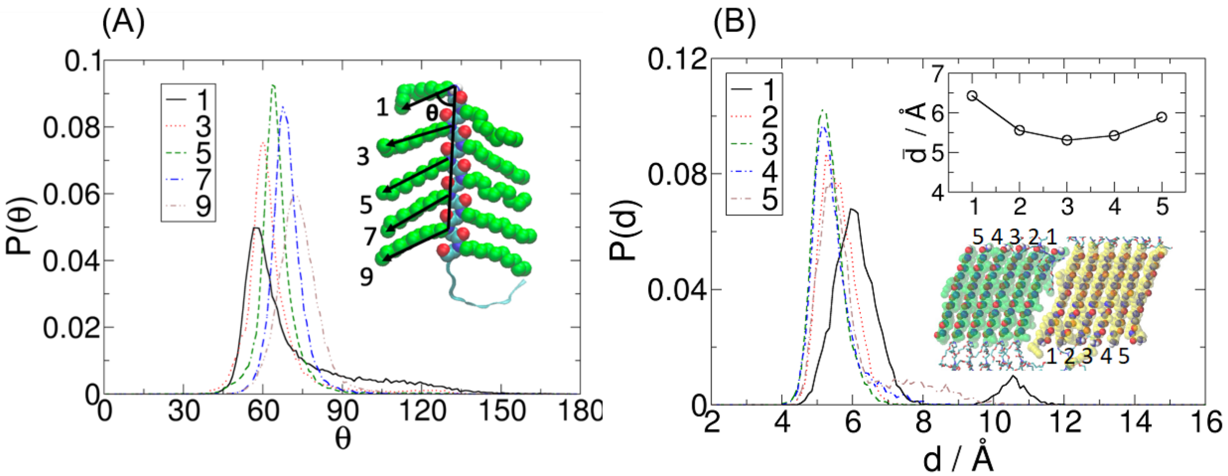

Figure S12. Quantitative evidence of less ordered solvent-exposed *N*-terminus. (A) Angle between the decyl side chain and backbone; (B) Distance between two neighboring *N*-termini in the same stack. The probability of θ > 90 indicates that it is possible to be off-lattice for the side chain numbered '1'. The larger distances between the neighboring molecules at the surfaces (labeled '1' and '5' in (B)) show the peel-off of the *N*-termini on the edges of each stack.

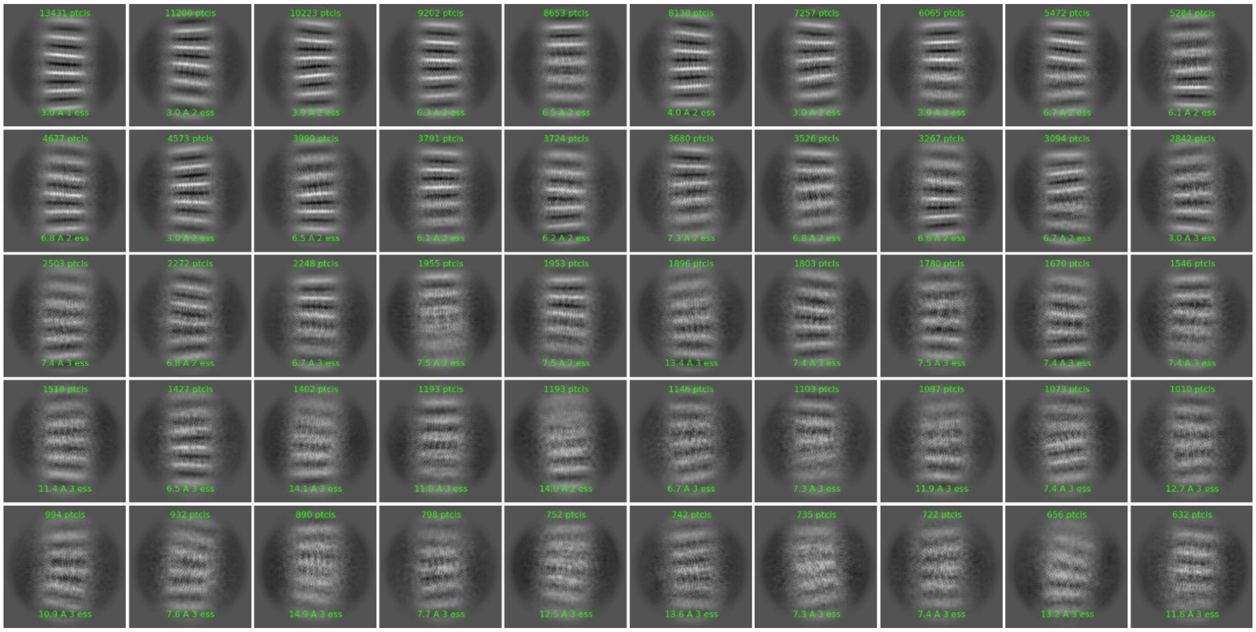

Figure S13. 2D classifications of the H-Ndc_{10}-Nte_{10} nanofibrils (sample **1**). Boxes were extracted along the fibers.

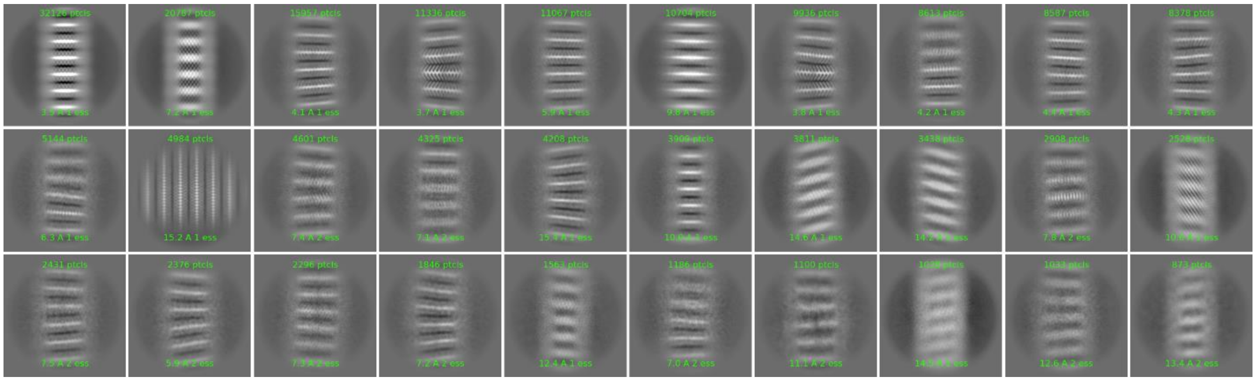

Figure S14. 2D classifications of the H-Ndc$_{10}$-Nte$_{10}$ nanofibrils (sample **2**). Box size is 256 pixels. Pixel size is 0.7 Å

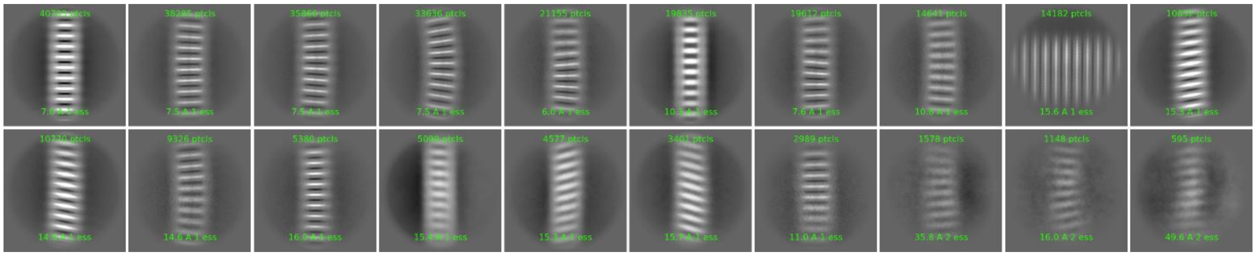

Figure S15. 2D classifications of the H-Ndc$_{10}$-Nte$_{10}$ nanofibrils (sample **2**). Box size is 400 pixels. Pixel size is 0.7 Å

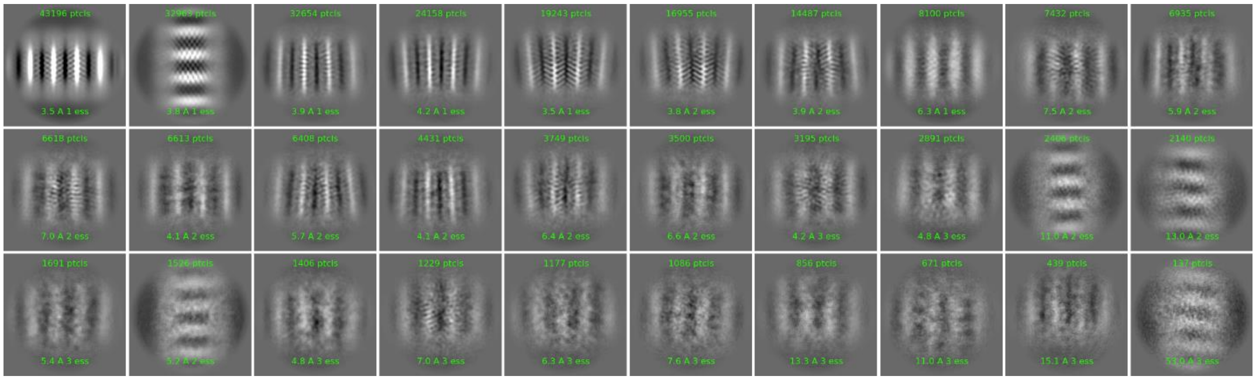

Figure S16. 2D classifications of the H-Ndc$_{10}$-Nte$_{10}$ nanofibrils (sample **2**). Box size is 180 pixels. Pixel size is 0.7 Å

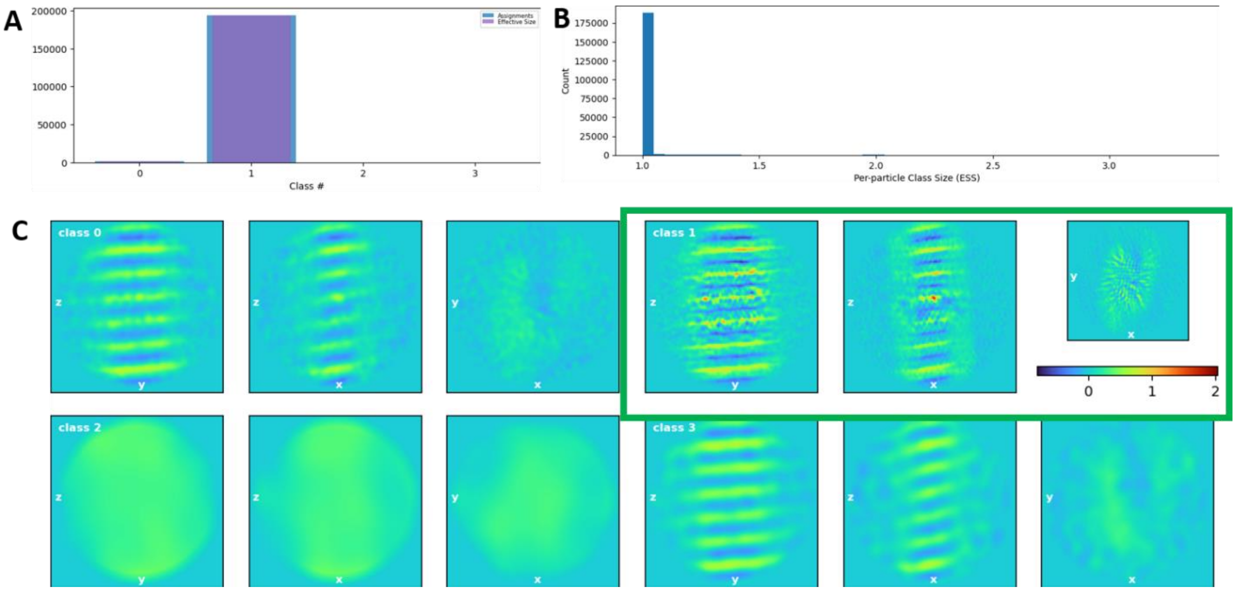

Figure S17. 3D classifications of the H-Ndc_{10}-b-Nte_{10} nanofibrils in urea solution (sample **2**): (A) class distributions, (B) probability distribution of classes. A value at 1 represents the presence of a single class, and (C) orthogonal views of 4 classes. The best class is indicated by the green box.

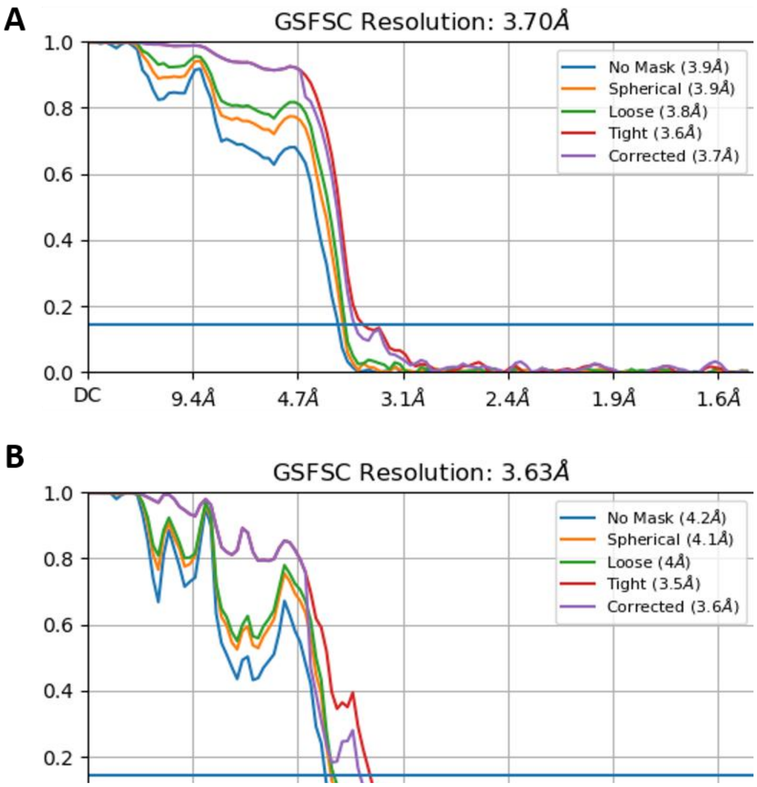

Figure S18. Fourier shell correlation measurements of (A) 3D reconstructed H-Ndc_{10}-Nte_{10} nanofibrils (sample **1**), and (B) H-Ndc_{10}-Nte_{10} nanofibrils in urea solutions (sample **2**). The straight line indicated the 0.143 criterion.

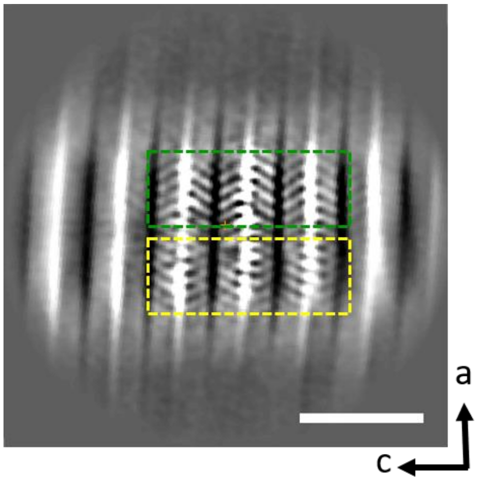

Figure S19. Averaged top view slices (thickness = 9.8Å) from the TEM 3D reconstruction of the nanofibril (sample **2).**

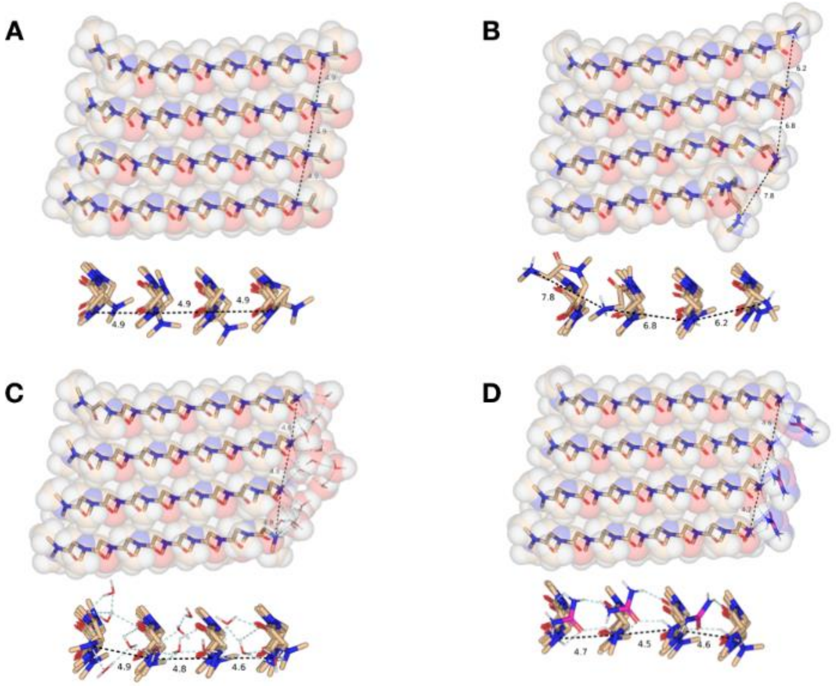

Figure S20. DFT models of different *N*-terminal environments on peptoid backbone packing in a minimal system: four (sarcosine)$_8$-NMe$_2$ oligomers aligned in parallel sigma strands. All models were optimized and converged at the M06-2X/6-31G* level of theory *in vacuo* using Gaussian16.[S1] Due to a tendency of poly-sarcosine oligomers to bend in these models, the distance between 2nd and 8th Calpha atoms in each oligomer was held fixed to preserve linearity, while allowing the *N*-terminal residue to adjust to the local environment. A) Uncharged acetyl caps show well-ordered *N*-termini, B) positively charged uncapped *N*-termini with no co-solvents show mutual repulsion and splaying, C) addition of 12 water molecules reduces splaying, D) addition of 3 urea molecules further tightens *N*-termini packing. Also notable is the number and regularity of hydrogen bonds each urea is capable of making between adjacent *N*-termini.

References

S1. M. J. Frisch, G. W. Trucks, H. B. Schlegel, G. E. Scuseria, M. A. Robb, J. R. Cheeseman, G. Scalmani, V. Barone, G. A. Petersson, H. Nakatsuji, X. Li, M. Caricato, A. V. Marenich, J. Bloino, B. G. Janesko, R. Gomperts, B. Mennucci, H. P. Hratchian, J. V. Ortiz, A. F. Izmaylov, J. L. Sonnenberg, D. Williams-Young, F. Ding, F. Lipparini, F. Egidi, J. Goings, B. Peng, A. Petrone, T. Henderson, D. Ranasinghe, V. G. Zakrzewski, J. Gao, N. Rega, G. Zheng, W. Liang, M. Hada, M. Ehara, K. Toyota, R. Fukuda, J. Hasegawa, M. Ishida, T. Nakajima, Y. Honda, O. Kitao, H. Nakai, T. Vreven, K. Throssell, J. A. Montgomery, Jr., J. E. Peralta, F. Ogliaro, M. J. Bearpark, J. J. Heyd, E. N. Brothers, K. N. Kudin, V. N. Staroverov, T. A. Keith, R. Kobayashi, J. Normand, K. Raghavachari, A. P. Rendell, J. C. Burant, S. S. Iyengar, J. Tomasi, M. Cossi, J. M. Millam, M. Klene, C. Adamo, R. Cammi, J. W. Ochterski, R. L. Martin, K. Morokuma, O. Farkas, J. B. Foresman, and D. J. Fox, Gaussian 16, Revision B.01, Gaussian, Inc., Wallingford CT, 2016.
